# Identification of Inhibitors with Potential Anti-Prostate Cancer Activity: A Chemoinformatics Approach

**DOI:** 10.3390/ph18060888

**Published:** 2025-06-13

**Authors:** Norberto S. Costa, Lúcio R. Lima, Jorddy N. Cruz, Igor V. F. Santos, Rai C. Silva, Alexandre A. Maciel, Elcimar S. Barros, Maracy L. D. S. Andrade, Ryan S. Ramos, Njogu M. Kimani, Alberto Aragón-Muriel, Juan M. Álvarez-Caballero, Joaquín M. Campos, Cleydson B. R. Santos

**Affiliations:** 1 Graduate Program in Pharmaceutical Sciences, Federal University of Amapá, Macapa 68902-280, AP, Brazil; nscquimica@gmail.com; 2 Laboratory of Modeling and Computational Chemistry, Department of Biological and Health Sciences, Federal University of Amapá, Macapa 68902-280, AP, Brazil; luciorolima@gmail.com (L.R.L.); jorddynevescruz@gmail.com (J.N.C.); raics@usp.br (R.C.S.); alexandremaciell@yahoo.com.br (A.A.M.); barrositb2008@hotmail.com (E.S.B.); mldsantosandrade@gmail.com (M.L.D.S.A.); ryanquimico@gmail.com (R.S.R.); 3 Graduate Program in Biotechnology and Biodiversity-Network BIONORTE, Federal University of Amapá, Macapa 68902-280, AP, Brazil; igorsantosvictor@gmail.com; 4 Natural Product Chemistry and Computational Drug Discovery Laboratory, University of Embu, Embu P.O. Box 6-60100, Kenya; njogu.mark@embuni.ac.ke; 5 Grupo de Investigaciones Bioquímicas, Universidad del Magdalena, Santa Marta CP 470004, Colombia; aaragonm@unimagdalena.edu.co; 6 Grupo de Química y Bioprospección de Productos Naturales, Departamento de Química, Universidad del Magdalena, Santa Marta CP 470004, Colombia; jalvarez@unimagdalena.edu.com; 7 Department of Pharmaceutical and Organic Chemistry, Faculty of Pharmacy, Campus of Cartuja, University of Granada, 18071 Granada, Spain; jmcampos@ugr.es; 8 Biosanitary Institute of Granada (ibs.GRANADA), University of Granada, 18071 Granada, Spain

**Keywords:** prostate cancer, antiandrogen, virtual screening, molecular docking, molecular dynamics

## Abstract

**Background:** Prostate cancer is the most common cancer in men, especially after the age of 50. It is a malignant disease that is increasing due to the increased life expectancy of the world population. Its development and progression are dependent on androgenic stimulation. **Objectives:** This study aimed to identify potential inhibitors with anti-prostate cancer activity through the application of chemoinformatics tools, exploring the Princeton (~1.2 million compounds) and Zinc Drug (~175 million compounds) databases. **Methods:** The methodology used several computational techniques, such as ROCS (Rapid Chemical Structure Superposition) and EON (Electrostatic Potential Screening), predictions of pharmacokinetic and toxicological properties, molecular docking, synthetic accessibility, biological activity, and molecular dynamics. **Results:** At the end of all these virtual screening steps, the study resulted in four promising potential candidates for the treatment of prostate cancer: the molecules ZINC34176694, ZINC03876158, ZINC04097308, and ZINC03977981, which exhibited all the desirable pharmacokinetic parameters (ADME/Tox) for a potential drug. **Conclusions:** Docking and molecular dynamics studies demonstrate stability and interaction with the androgen receptor of the selected compounds, showing them to be promising candidates for the development of new drugs.

## 1. Introduction

Prostate cancer (PCa) is the second most common cancer in men worldwide and is one of the main causes of cancer mortality in the male population [1]. The number of people diagnosed with prostate cancer will more than double worldwide over the next 2 decades, from 1.4 million in 2020 to 2.9 million in 2040, according to findings from a Lancet Commission. More people will also die from the disease, with an estimated 700,000 patients expected to die by 2040, compared with 375,000 people in 2020 [2].

Androgen deprivation therapy (ADT) and androgen receptor (AR) inhibitors are the mainstay of treatment, especially in advanced stages [3]. However, resistance to treatments has driven the search for new therapeutic strategies, both experimental and computational, to overcome tumor escape mechanisms [4,5].

The androgen receptor (AR) remains a crucial therapeutic target in prostate cancer, especially in castration-resistant prostate cancer (CRPC). Resistance to conventional treatments fuels the demand for innovative inhibitor designs [6,7]. The AR inhibitor landscape is rapidly evolving, with computational tools (AI, dynamic coupling) enabling precision design and experimental therapies (PROTACs, NTD inhibitors) addressing resistance [8]. Clinical successes in triple therapies and PARP combos highlight the shift toward multi-targeted regimens, while computational advances promise to accelerate the discovery of next-generation AR degraders and allosteric inhibitors [9].

Huggins and Hodges (1941) first reported androgen deprivation therapy (ADT) as the first hormonal therapy for prostate cancer [10]. Any medication that suppresses androgenic activity is included in ADT. This can be done in two ways: either by employing antiandrogens to prevent AR signaling (AR deactivation) or by reducing testicular and/or extragonadal androgen production through medical or surgical castration (i.e., AR ligand reduction). Complete androgen blocking is the term used to describe the combination of both strategies [11].

Antiandrogens compete with these androgens for binding sites on androgen receptors in the prostate cell nucleus, thus promoting apoptosis (regulated cell death) and inhibiting the growth of prostate cancer [12]. Thus, once the patient is subjected to androgen suppression, a regression in prostate cancer is observed [13].

In terms of structure, they can be separated into nonsteroidal and steroidal antiandrogens. Differentiating between the biological effects of steroids and nonsteroidal drugs is based on how they affect blood testosterone levels and how active they are at receptors other than the androgen receptor [14].

**Figure 1:**
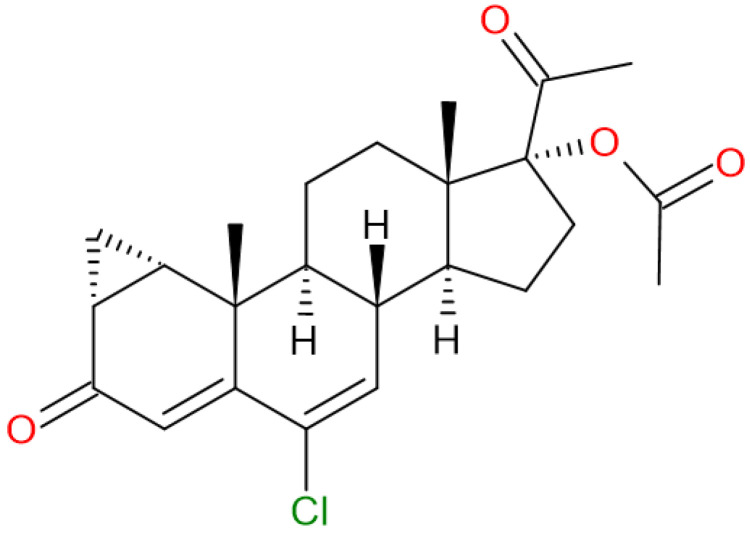
Structural formula of cyproterone acetate (CPA).

Nonsteroidal antiandrogens can work in several ways to block the effects of androgens. For example, some nonsteroidal antiandrogens can inhibit the production of androgens by suppressing the activity of enzymes involved in their synthesis [15]. Others may directly block androgen receptors or interfere with cell signaling pathways that are activated by androgens. Regarding apalutamide, darolutamide and enzalutamide are new generation anti-androgens that not only interfere with AR translocation to the nucleus and obstruct AR-mediated transcription, but also more brightly and competitively inhibit the AR ligand-binding domain than first generation agents [16]. Cyproterone acetate (CPA; Figure 1) was the first antiandrogen that was used to treat advanced prostate cancer. It is a synthetic steroid, which has strong antiandrogenic activity and weak progestational activity. The weak progestational activity of CPA produces an anti-gonadotropic action on the hypothalamic-pituitary-gonadal (HPG) axis [17].

CPA is mainly an inhibitor of the interaction between androgen and androgen receptors. Furthermore, it was originally utilized to treat advanced prostate cancer because of its capacity to block the androgen receptor (AR) and thereby lower serum testosterone levels [18]. In prostate cells, CPA inhibits the binding of dihydrotestosterone to androgen receptors, thus reducing effective plasma testosterone levels. Numerous investigations have clearly demonstrated the testosterone-suppressive effectiveness of CPA, particularly at high dosages (50 mg/day) [19].

Although the CPA molecule has lower binding affinity (ΔG) than second-generation non-steroidal antiandrogens such as Enzalutamide and Apalutamide [19], it was chosen as the reference molecule in this study because it is complexed in the AR and deposited in the Protein Database at the crystallographic position with the PDB code-2OZ7 (https://doi.org/10.2210/pdb2oz7/pdb; accessed on 15 August 2023) [20].

Because virtual screening (VS) approaches use known molecules and save time and money, they have been studied to find possible candidates for the therapy of many diseases [21]. For this process, it is essential to use a number of databases that act as a library of chemical compounds. These databases are searched based on particular parameters, such as electrostatic similarities and overlapping chemical structures [22]. The exponential rise in computational resources and the ongoing advancement of simulation and machine-learning methods have made VS a widely used tool for molecular discovery [23].

**Figure 2:**
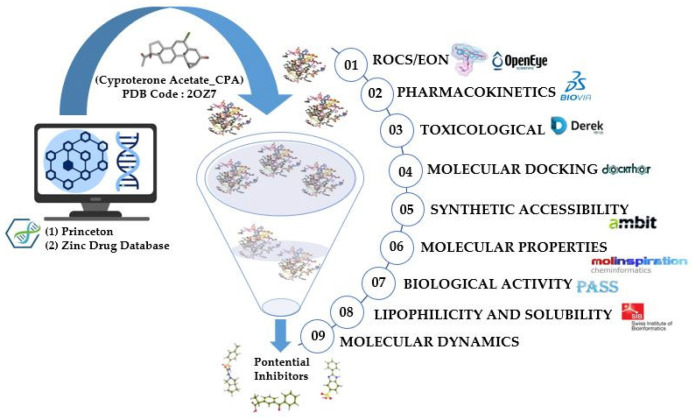
Methodological steps of the research.

In general, this article employs chemoinformatics strategies to identify new compounds with the potential to inhibit the androgen receptor. ROCS and EON filtering techniques were used in the Princeton (https://chemistry.princeton.edu/research-facilites/small-molecule-screening-center/; accessed on 22 September 2023) and Zinc Drug [24] databases, with cyproterone acetate as a comparison molecule. The selected compounds were evaluated considering their physicochemical and toxicological characteristics. The molecules that obtained the best results were subjected to molecular docking to analyze their molecular interactions and their potential as drug candidates. Figure 2 illustrates the steps adopted in the methodological system of this research.

## 2. Results

### 2.1. Rapid Overlay of Chemical Structures (ROCS) and Electrostatic Similarity (EON)

Finding comparable compounds chemically is a key strategy in ligand-based drug development. Finding and returning database compounds with the model compound’s structure and bioactivities is its aim. A fundamental premise of similarity-based virtual screening is the idea of chemical similarity, which asserts that molecules with similar structures are likely to have similar bioactivities [25].

Using the OpenEye Rapid Overlay of Chemical Structures (ROCS) program (available at https://www.eyesopen.com/rocs; accessed on 5 October 2023) enabled ligand-based virtual screening through conformational comparison of the volume of the reference structure. The technique of Gaussian functions found in atoms is used for this categorization; it suggests the optimal overlap between molecules in a collection of features, which may include a steric volume or a molecular interaction known as Comboscore [26].

The CPA molecule was utilized in this study’s ROCS program as a comparison model with every other molecule in the Princeton and Zinc Drug databases. The form “Top2000/base” was employed to assess the molecules’ similarity, and this produced 4000 structures. The EON program’s input files were created by this software [27].

Starting from the structures previously aligned by the ROCS program (version 3.4.2), the electrostatic similarity of the structures was calculated by the EON program (version 2.3.2) (https://www.eyesopen.com/eon; accessed on 18 October 2023) using the same reference structure (CPA). EON selects compounds with comparable morphologies and electrostatic potential by comparing the electrostatic potential maps of pre-aligned molecules and determining the Tanimoto index measurements for comparison [28]. The screening process resulted in the selection of the “Top500/base”.

### 2.2. Predictions of Pharmacokinetic Properties

**Figure 3:**
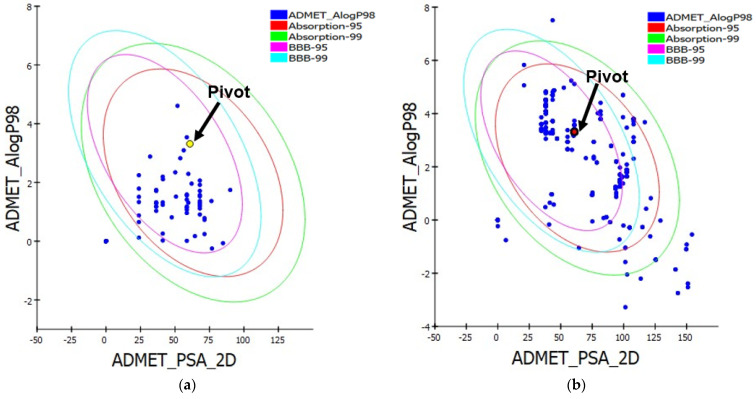
Pharmacokinetic property charts: (**a**) Princeton Database and (**b**) Zinc Drug Database. Polar surface area (PSA) against AlogP graphic illustrating molecules in the intestinal absorption (HIA) and blood-brain barrier (BBB) 95% and 99% confidence limit ellipses. Cyproterone Acetate Pivot (CPA). The yellow and red dots correspond to the pivot molecule (reference) in the study.

The 1000 molecules resulting from the screening process in the ROCS and EON stages were subjected to the prediction of pharmacokinetic properties: absorption, distribution, metabolism, and excretion (ADME) using the Biovia Discovery Studio Visualizer program (version 21.1.0.202982) (https://discover.3ds.com/discovery-studio-visualizer-download; accessed on 15 December 2023). Based on 95% and 99% confidence values for the blood-brain barrier (BBB), human intestinal absorption (HIA), polar surface area (PSA), and lipid solubility (LogP), the graphs in Figure 3 were created [29].

The molecule that served as the pivot (reference) for the pharmacological properties is CPA, which is indicated in the graphs. Promising candidates’ 95% and 99% confidence intervals are displayed as ellipses. The compounds detected in this area resemble those with an absorption capacity of at least 90%, a PSA value of less than 140, and an ALogP98 value of less than 5. Candidates are only chosen if they fall within the 95% to 99% confidence interval [29].

Table 1 displays the outcomes of the ADME predictions. The evaluation of the 24 compounds chosen in conjunction with the CPA was conducted using the following descriptors: CYP2D6 Binding, CYP3A4 Binding (http://www.swissadme.ch/; accessed on 13 November 2024), Human Intestinal Absorption, Aqueous Solubility, Blood-Brain Barrier Penetration, and Plasma Protein Binding.

Predictive analysis revealed that none of the evaluated molecules demonstrated inhibitory potential for CYP2D6, a favorable result considering the critical role of this enzyme in the metabolism of drugs such as antidepressants and antipsychotics [30]. However, three compounds (ZINC3688590, ZINC34176695, and ZINC34176694) showed inhibitory activity for CYP3A4. This finding is relevant, since CYP3A4 metabolizes approximately 50% of marketed drugs, and its inhibition can increase the plasma levels of other drugs metabolized by this enzyme, leading to toxicity [31]. Compounds that inhibit CYP3A4 are often discarded in preclinical screenings to avoid regulatory issues [32]. The ability of a drug to inhibit or not CYP3A4 is vital for therapeutic safety and efficacy, impacting clinical and pharmaceutical decisions. The majority of molecules (83%) showed good solubility, and all had excellent results for intestinal absorption, parameters responsible for the transport and release of the drug.

One well-known phenomenon is the binding of medications and metabolites to plasma proteins [33]. Small molecules, such as chemicals and medications, have the ability to attach to blood plasma proteins. The two main plasma proteins that bind pharmaceuticals are albumin and α1-acid glycoprotein (α1-AGP); lipoproteins also play a significant role, especially for drugs that are extremely lipophilic; however, γ, thyroid, and steroid-binding globulins appear to have a smaller effect [34].

Owing to their high concentration, plasma proteins efficiently attenuate drug potency in vivo by controlling the concentration of free drug in both plasma and compartments in equilibrium with plasma [33]. All the molecules analyzed, as well as the reference (CPA), presented false negative results for binding to plasma proteins. This is explained by the low percentage of binding of these molecules to these proteins (see Supplementary Material—Table S1).

The brain is shielded from dangerous substances by the semi-permeable blood-brain barrier, which can also restrict the entry of drugs [35]. The results obtained demonstrate that the majority of molecules have low penetration into the BBB, which is satisfactory for the target (Androgen receptor).

### 2.3. Predictions of Toxicological Properties

**Figure 4:**
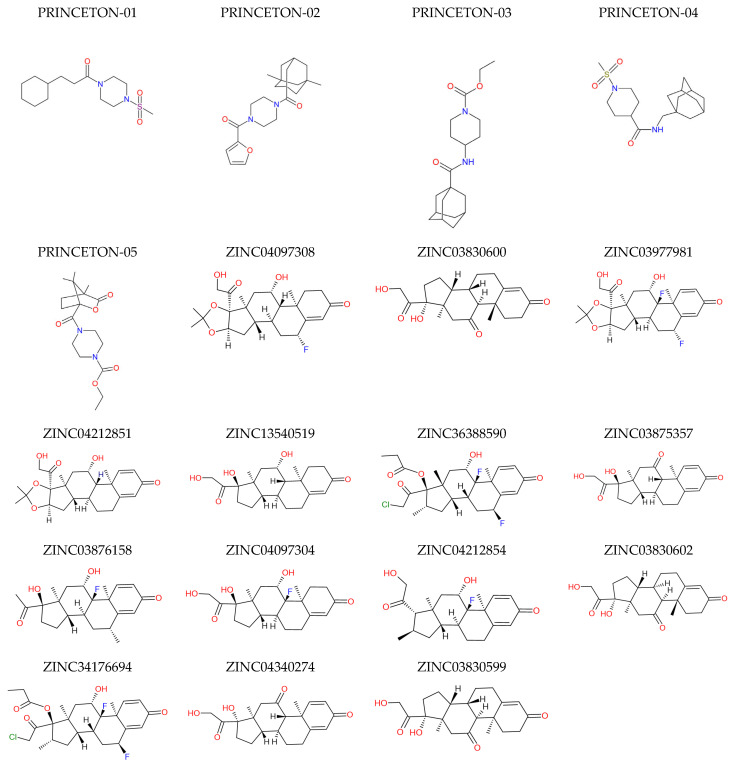
Molecules selected for molecular docking study.

The DEREK 10.0.2 Nexus program was used to carry out the in silico toxicological investigation. Due to the presence of toxicophoric groups in the compounds, toxicity alerts were confirmed at this point in the study and generated alerts that needed further investigation. The Toxicity Class was obtained through the Protox II online server, as well as the Lethal Dose value (LD_50_) in mg·kg^−1^ [36]. These parameters were fundamental for the selection of the most promising compounds in this study. In this step, structures without warnings or that were in the toxicological class IV–VI range and presented fewer warnings of possible toxicity when compared to the reference compound were selected (see Supplementary Materials—Table S2). This resulted in 19 molecules (Figure 4) that were subjected to a molecular docking study.

### 2.4. Molecular Docking

Preceding toxicological analysis, 19 molecules were selected for the molecular docking stage. To find out if the program can accurately predict the location of the ligand in the protein’s active site, redocking was required to validate the molecular docking procedure utilized in this study. This process consists of comparing the position of a co-crystallized ligand (Cyproterone Acetate-PDB code 2OZ7) with the position of a ligand anchored to the active site of the same protein. It was carried out for comparison through the structural overlap between the original structure, which contains the protein (Androgen Receptor) and the 2OZ7 inhibitor, with the result obtained in the redocking simulation using the DockThor (version 2.0) server (https://www.dockthor.lncc.br/v2/; accessed on 19 February 2024) [37].

**Figure 5:**
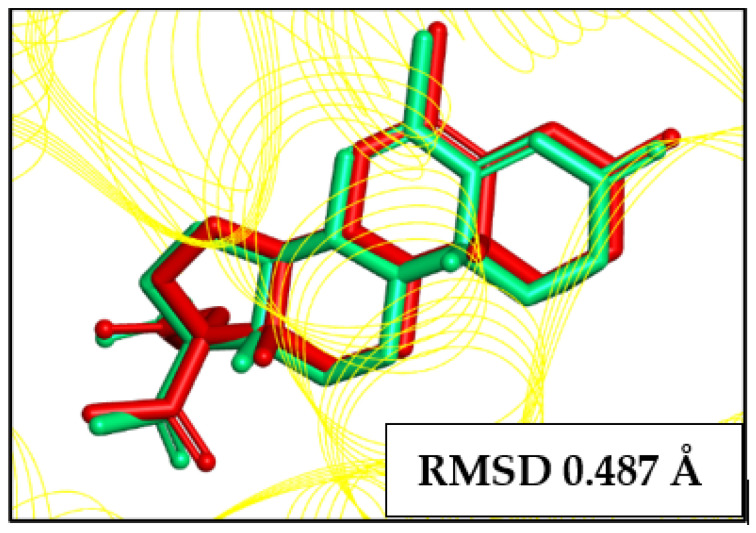
Validation of the molecular docking protocol: a comparison of the optimal conformation obtained from the computer simulation (red) and the crystallographic ligand (green).

The molecular docking validation yielded satisfactory findings, as seen by the identical relative locations of the anchored and crystallographic ligands (Figure 5). The methodology was validated by the RMSD of 0.487 Å that was acquired during the optimal position redocking process [38].

**Figure 6:**
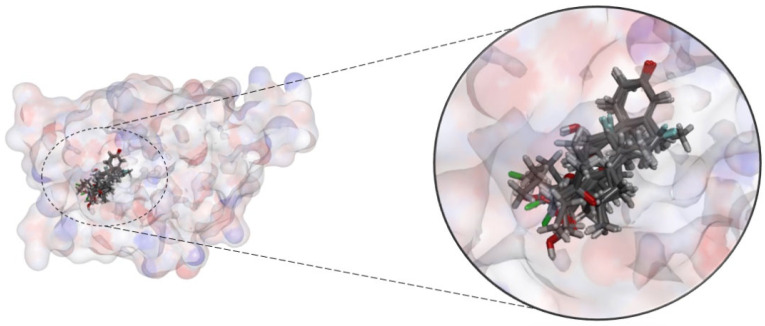
Molecules docked in the AR active site.

After process validation, the 19 molecules selected in the toxicological analysis were subjected to the molecular docking test (Figure 6) through the DockThor server following the same parameters used in redocking.

Thus far, four interesting compounds have been identified, as indicated in Table 2 (see Supplementary Material—Table S3), based on binding affinity values (ΔG) and interactions with the primary amino acid residues relative to the reference molecule.

The reference molecule showed a binding affinity of −10.827 kcal mol^−1^ with the androgen receptor interacting with two amino acid residues-Asn705, Arg752-through hydrogen bonds. Hydrogen bonds are of great importance for the stability of the ligand in the protein, playing an important role in its binding affinity [39]. Acyl, π-alkyl, and van der Walls interactions also occur, which interact in a hydrophobic way (Figure S1, see Supplementary Materials).

The best binding affinity value occurred for the ZINC34176694 molecule, obtaining values of −10.850 kcal mol^−1^, higher than the pivot molecule; however, for this ligand there was only one hydrogen bond between the carbonyl oxygen and the Arg752 residue (Figure S2, see Supplementary Materials). The main amino acid residues are present (Leu701, Leu704, Met780, Phe876, and Ser778) [40]. These interactions are hydrophobic of the alkyl, π-alkyl, and van der Waals types. They represent a similarity of 87.5% with the reference molecule, demonstrating a very satisfactory result.

The ligand ZINC03876158 had a binding affinity value of −10.369 kcal mol^−1^, showing a hydrogen interaction with the amino acid residue Met780. They interact with the amino acid residues Leu701, Leu704, Leu880, Phe876 in a hydrophobic way. The percentage of similarity of interactions of the amino acids present in the ZINC03876158 ligand in relation to the amino acids that interact in the reference molecule is 91.7%, which represents an excellent result for a potential drug candidate (Figure S3, see Supplementary Materials).

For the ligand ZINC04097308, three hydrogen bonds are observed (Figure S4, see Supplementary Materials) with the amino acid residues-Ala877, Arg752, Asn705-showing a binding affinity of −10.260 kcal mol^−1^. All other interactions are hydrophobic, presenting the main amino acid residues (Leu701, Leu704, Met780, Phe876, and Ser778). This ligand showed 100% similarity with the reference molecule in relation to the amino acid residues present, which represents a very promising result for the following steps.

The binding affinity of the ligand ZINC03977981 was −10.237 kcal mol^−1^, generating a hydrogen bond with the amino acid residue Asn705. The main interactions with AR (Leu701, Leu704, Met780, Phe876, and Ser778) are present. This ligand also showed 100% similarity of interactions with the amino acid residues that the reference molecule presents (Figure S5, see Supplementary Materials), which qualifies it for the other stages of the research.

### 2.5. Synthetic Accessibility Prediction

After the molecular docking evaluation, the 4 selected molecules underwent the synthetic accessibility test (SA), aiming to verify those that have the greatest probability of being synthesized. SA prediction was done using AMBIT-SA (version 3.1.0) and SwissADME software (version 4.0). In the AMBIT-SA software, an algorithm calculates several parameters related to the chemical characteristics of the structures and scores from 0 to 100, where the value 100 refers to a molecule that is very easy to synthesize [41].

In contrast, SwissADME is primarily based on the idea that the ease of synthesis correlates with the frequency of molecular fragments in the ‘really’ achievable molecules. For frequent chemical fractions, the fragmented contribution to SA should be favorable; for rare fractions, it should be unfavorable. Normalized values are between 1 (very easy) and 10 (extremely difficult to synthesize) [42].

Cyproterone acetate (CPA), chosen as a standard for comparison, exhibited medium synthetic accessibility based on its SA values: 23.712 in AMBIT-SA and 5.54 in SwissADME, which both designate it as a medium-complexity molecule to synthesize (Table 3). In comparison to the reference molecule, ZINC03876158 was projected by the AMBIT-SA software to have a score of 31.245, indicating the greatest deviation (±7.533). This suggests a potentially unique profile for this candidate compared to the reference. The smallest variation occurred with the ZINC34176694 molecule (Score 23.097) of ±0.615, suggesting a greater similarity to the reference.

In the SwissADME software, all the molecules analyzed had SA values very close to the reference molecule, with an average variation of ±0.33. This is probably due to the similarity in their molecular scaffolds, which are mainly composed of 4 rings. In both software packages, the molecules were classified as having similar average synthetic accessibility to the reference molecule. Therefore, no molecules were discarded for the next stage of research.

### 2.6. Predictions of Molecular Properties

In the analysis of molecular properties, the theoretical oral bioavailability of specific molecules in synthetic accessibility was evaluated using Lipinski’s “Rule of Five-RO5” as a parameter. According to the findings of Lipinski et al. [43], drug-like compounds must adhere to RO5, which means that multiple violations are not permitted.

These requirements include a molecular weight (MW) ≤ 500 Da, a number of hydrogen bond donors (HBD) ≤ 5, a number of hydrogen bond acceptors (HBA) ≤ 10 and an octanol-water splitting coefficient (Log P) ≤ 5 [42]. For this prediction, the Molinspiration Cheinformatics program (version 2021.10) was used (https://molinspiration.com/cgi/properties; accessed on 10 March 2024). The results obtained are presented in Table 4.

All of the chosen compounds’ HBA, HBD, LogP, and MW values, as displayed in Table 4, fall within the permissible range, as indicated by RO5. Thus, like the reference molecule, none of the molecules analyzed violated Lipinski’s rule, which predicts that they have good oral bioavailability. These results theoretically demonstrate that these molecules will have a good distribution throughout the body, enabling greater capacity to reach the target organ and execute their therapeutic and pharmacological effect. Topological polar surface area (TPSA) is known to be a reliable measure of blood-brain barrier penetration (TPSA less than 60 Å^2^) and intestinal medication absorption (TPSA less than 140 Å^2^) [44]. This parameter predicts the cellular permeation capacity of chemical compounds; it is therefore assumed that the lower the TPSA value the better. Therefore, it is suggested that increasing TPSA decreases the transport capacity of drugs, which in turn affects their biological activities [44].

Compounds present computational TPSA values between 74.60 and 93.07 Å^2^, which predicts good intestinal absorption. However, all the molecules analyzed do not present adequate penetration of the blood-brain barrier (BBB), as the TPSA values are higher than 60 Å^2^, which confirms them as potential candidates for PCa inhibitors. These properties are directly related to molecular pharmacokinetics, implying that these molecules can become strong candidates for a drug. With the help of the SwissADME software, the Bioavailability Radar graph was determined (Figure S6, see Supplementary Materials), responsible for presenting a set of properties designed for excellent oral bioavailability, through information such as molecule size, flexibility, solubility, lipophilicity, saturation, and polarity. The area marked in pink has the function of delimiting the ideal bioavailability conditions for oral administration of a medication [45].

All molecules analyzed theoretically show excellence for oral bioavailability, since all physicochemical parameters evaluated are within the pink area, which represent satisfactory bioavailability standards (Figure S7, see Supplementary Materials). The probability of the molecule being orally bioactive increases with the number of factors that fall within the optimal range [45].

### 2.7. Biological Activity Prediction

The PASS online web server (version 2.0) (www.pharmaexpert.ru/passonline; accessed on 19 March 2024) was used to predict the biological activities of the chosen compounds and the reference molecule (CPA). PASS calculates the probability of being inactive (Pi) and active (Pa) values using the molecules SMILES code. A compound’s biological activity can be determined by looking at its Pa and Pi values.

The biological activities that were chosen had some connection to anticancer activity. If Pa is more than 0.7, the small molecule is expected to be very active in experiments; if Pa is between 0.7 and 0.5, moderate activity is predicted; and if Pa is less than 0.5, the biological effect is expected to be insignificant [45].

The prediction of the biological activities of the four molecules obtained in the screening process presented values of activities related to cancer (Table 5), with the molecule ZINC03876158 presenting prediction values for the Androgen Antagonist higher than the reference molecule (CPA). It is also worth highlighting the molecule ZINC34176694, which presented a Pa value > 0.7. These two molecules demonstrate satisfactory values to be considered potential prostate cancer inhibitors.

The molecules ZINC04097308 and ZINC03977981 presented Pa values < 0.5 for androgen antagonist, which predicts minimal biological activity [46]. As all molecules showed activities related to cancer, especially prostate cancer, even if some values were lower than the reference molecule, they were considered for the next stage of the research.

### 2.8. Predictions of Lipophilicity and Water Solubility

The lipophilicity and water solubility properties of the selected compounds were determined using the SwissADME website. These properties are important in the search for new drugs, as they influence the kinetics of the action of the therapeutic candidates. Lipophilicity is measured by the octanol-water partition coefficient (LogPo/w), which is derived from the concentration of a neutral molecular form in the organic and aqueous phases [47]. Lipophilicity is the most important of all physicochemical properties measured and tracked in a chemical optimization program for a drug candidate because it affects solubility, ligand-target binding interactions, ADME properties (absorption, distribution, metabolism, and excretion), in vivo toxicological results, and ultimately the overall quality of the drug candidate [48].

The SwissADME server offers five publicly available models (XLOGP3, WLOGP, MLOGP, SILICOS-IT, and iLOGP) for evaluating the lipophilicity of a molecule. XLOGP3 predicts the log P value of a query compound by starting with the known log P value of a reference compound and applying an atomistic technique with two correction factors and 87 different types of atoms; a fragmentary system serves as the basis for the purely atomistic WLOGP method [49], while MLOGP is a topological method model based on a linear relationship with 13 implemented molecular descriptors [50]. SILICOS-IT is a hybrid method that combines 27 fragments and 7 topological descriptors, and iLOGP is a physically based method that uses the accessible surface area of the solvent (GB/SA) and the free solvation energies of n-octanol and water as well as the generalized implicit Born solvent equation.

The SwissADME server analyzed the four selected structures together with the reference molecule to determine their lipophilicity values and an average of the results (Table 6; see Supplementary Materials—Figure S8). All predicted molecules presented values below 5, as proposed by Lipinski for compounds with desirable lipophilicity [43]; none of the analyzed molecules presented an average higher than the pivot. These investigations yielded values ranging from 1.38 to 4.89, indicating a high lipophilicity and satisfying the necessary requirements for potential therapeutic candidates.

It is worth highlighting the molecules ZINC03876158, ZINC04097308, and ZINC03977981 that presented an average Log P within the range 0 < LogP < 3, which is considered an optimal range for better permeability of a drug, where several studies have demonstrated that 99% of the compounds that exhibit more than 80% oral bioavailability fall within this range [51].

Water solubility (LogS) plays an essential role in chemistry, life, and the development of new drug candidates. As it will influence the toxicity, bioavailability, and absorption of these candidates. Therefore, it is one of the most important pharmacokinetic properties during the drug development process [52].

For early-stage drug discovery, predicting solubility is particularly crucial since insoluble compounds could not be available for biochemical testing. These factors have led to a significant use of in silico solubility prediction [52].

Three topological approaches for water solubility are available via the SwissADME web server: the ESOL method, the Ali method, and the SILICOS-IT method [53]. According to Table 7 (Figure S9, see Supplementary Materials), the molecules’ LogS values for the ESOL technique ranged from −4.88 to −3.28, the Ali method from −5.14 to −2.94, and the SILICO-IT method from −5.00 to −3.37. As the LogS values range from −4 to −6, the ZINC34176694 molecule demonstrated moderate solubility (consensus LogS −5.01), indicating that its solubilization in water is only conceivable with organic solvents, in line with the reference molecule (consensus LogS −4.63) [54].

The molecules ZINC03876158, ZINC04097308, and ZINC03977981 show good solubility, as the average LogS values are in the −2 to −4 range, demonstrating that these molecules are theoretically soluble in biological fluids due to their solubility values (LogS > −5). These data are essential for future in vitro tests aimed at validating the computational methods used in this study [55,56].

### 2.9. Molecular Dynamics Simulation

**Figure 7:**
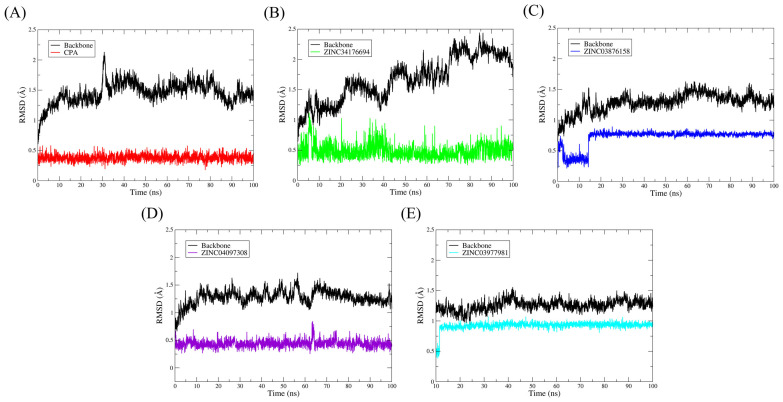
Analysis of the conformational stability of the systems over 100 ns of MD simulation. The protein backbone is represented in black in all graphs, while the colors representing the binders vary. The RMSD graphs were plotted in relation to the systems obtained after the steps of minimization, heating, and equilibrium. (**A**) AR-CPA, (**B**) AR-ZINC34176694, (**C**) AR-ZINC03876158, (**D**) AR-ZINC04097308, and (**E**) AR-ZINC03977981.

Molecular dynamics (MD) simulations are essential tools for exploring the temporal behavior of biomolecular complexes at the atomic level, enabling the assessment of structural stability and the persistence of intermolecular interactions over time. In this study, 100 ns simulations were performed for five complexes formed between the androgen receptor (AR) and selected ligands, aiming to validate and refine results previously obtained through molecular docking, see Figure 7.

The root-mean-square deviation (RMSD) of the protein backbone was used as the primary metric to monitor the overall structural stability of each system. As illustrated in Figure 7, all complexes—AR–CPA, AR–ZINC34176694, AR–ZINC03876158, AR–ZINC04097308, and AR–ZINC03977981—exhibited mild fluctuations and stabilization within the initial nanoseconds, indicating structural convergence and the absence of ligand detachment events throughout the simulation period.

The low RMSD values indicate that the ligands remained anchored in the AR binding site, maintaining relevant contacts with key residues. This behavior reflects a strong steric and electronic complementarity between the ligands and the binding cavity, which is a preliminary indicator of potential modulatory activity. Figure 7 presents RMSD profiles after energy minimization, gradual heating, and equilibration stages, with the protein backbone shown in black and each ligand in distinct colors. These visual representations allow for direct comparisons among the simulated systems and facilitate the identification of stability patterns.

In addition to global conformational analysis, interaction profiling revealed persistent contacts between ligands and critical residues, particularly Leu701, Leu704, Met780, and Phe876. Notably, compounds ZINC34176694 and ZINC03876158 exhibited strong hydrophobic and van der Waals interactions, consistent with the predominantly hydrophobic nature of the AR binding pocket as described for both endogenous and synthetic ligands.

Moreover, ZINC03876158 and ZINC03977981 formed stable electrostatic interactions and hydrogen bonds with Arg752 and Met780, suggesting an anchoring mechanism supported by both hydrophobic and polar contributions. Literature evidence highlights this dual interaction profile as crucial for the selectivity of AR modulators.

Residue-level analysis throughout the simulations confirmed the consistent involvement of Asn705, Arg752, and Met780 in ligand recognition and stabilization, designating them as structural hotspots. These residues represent strategic targets for future rational modifications aimed at enhancing affinity and specificity.

Overall, MD simulations not only validated the docking results but also offered dynamic insights into ligand behavior, reinforcing the potential of the investigated compounds as selective androgen receptor modulators.

The analysis of the structural fluctuations of the target proteins during the molecular dynamics simulations was conducted based on the root mean square deviation (RMSF) and radius of gyration (Rg) plots. These metrics are crucial for understanding the local flexibility of the residues and the global conformational stability of the protein-ligand complexes.

**Figure 8:**
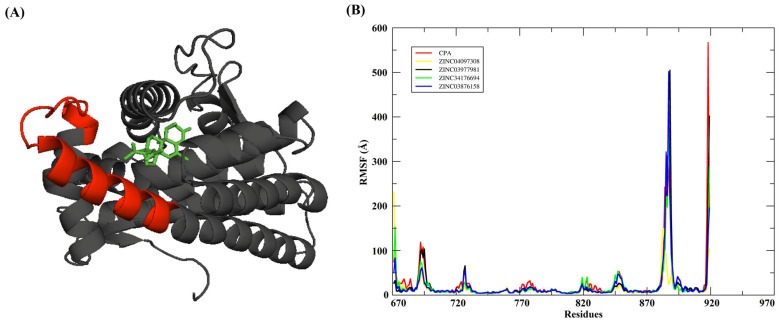
(**A**) Region of the protein that showed the greatest RMSF fluctuation. (**B**) Overlapping of RMSF graphs of the protein backbone.

As illustrated in Figure 8A, the Root Mean Square Fluctuation (RMSF) analysis reveals that the regions with the highest atomic fluctuations are predominantly located at the terminal portions of the protein and in solvent-exposed loop regions. This is consistent with typical molecular dynamics behavior, where these segments possess fewer structural constraints and greater conformational flexibility. In particular, the region encompassing residues approximately between 890 and 915 exhibited the most pronounced fluctuations, indicating intrinsic mobility. This flexibility may play an essential role in molecular recognition, ligand accommodation, or in facilitating conformational changes necessary for the protein’s functional activity.

The RMSF profiles shown in Figure 8B demonstrate that, despite the presence of chemically diverse ligands, the overall dynamic behavior of the protein remains highly conserved across all complexes. This indicates that ligand binding does not significantly perturb the global structural dynamics of the protein, as the core regions stabilized by secondary structural elements, such as α-helices and β-sheets, consistently maintain low RMSF values throughout the simulations. However, subtle yet meaningful local variations are evident between different complexes, suggesting that some ligands are capable of promoting enhanced stabilization of specific protein regions. This likely occurs through the formation of additional non-covalent interactions, including hydrogen bonds, hydrophobic contacts, salt bridges, and π-π stacking, and may also involve allosteric effects where binding influences the flexibility of regions distant from the active site.

In summary, the RMSF analysis provides critical insights into the residue-level dynamics of the protein-ligand complexes, revealing how ligand binding influences both global stability and local flexibility. While the general conformational integrity of the protein remains intact, certain ligands demonstrate the ability to reduce the mobility of key regions, which could enhance binding affinity, specificity, and inhibitory efficiency. These findings underscore the importance of integrating dynamic evaluations such as RMSF into drug discovery pipelines, as they contribute valuable information about the impact of ligand binding on protein behavior, supporting the rational design and optimization of more effective therapeutic candidates.

**Figure 9:**
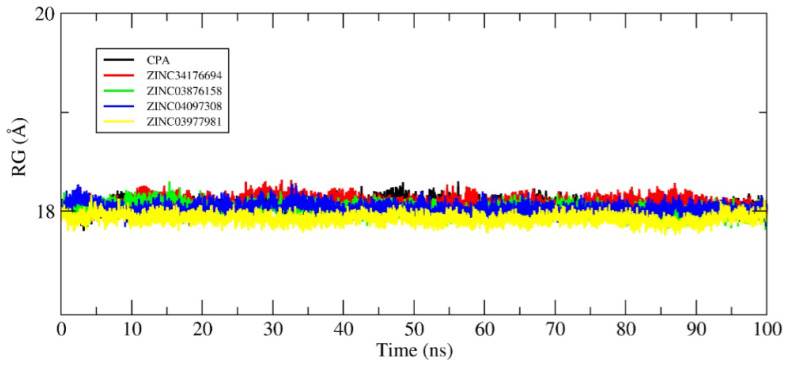
Conformational analysis radius of gyration of all biomolecular complexes.

Figure 9 presents the analysis of the radius of gyration (Rg), a critical parameter for evaluating the overall compactness and structural stability of the protein-ligand complexes throughout the molecular dynamics simulations. The Rg measures how the mass of the protein is distributed relative to its center of mass, serving as an indicator of whether the protein maintains its native folded state or undergoes conformational changes over time. Monitoring Rg is essential for detecting potential unfolding events or destabilizations that may occur upon ligand binding.

The results demonstrate that all protein-ligand complexes maintained relatively stable Rg values throughout the 100 ns of simulation, with fluctuations confined within a narrow range. This stability indicates that ligand binding did not induce significant global structural perturbations or the unfolding of the protein. The preservation of the Rg within consistent limits suggests that the overall tertiary structure of the protein remains compact and well-organized, regardless of the ligand interacting with it. Notably, the complexes involving certain ligands exhibited slightly lower Rg fluctuations, suggesting enhanced conformational stability.

In this context, complexes that showed smaller variations in Rg can be considered more structurally stable, which is a highly desirable property when evaluating potential molecular inhibitors. Ligands capable of maintaining or enhancing the structural rigidity of the target protein may contribute to more stable binding interactions and potentially improved inhibitory efficacy. Therefore, the Rg analysis not only confirms the absence of destabilizing effects induced by the ligands, but also highlights subtle differences in stabilization capacity among the tested compounds, an important factor to consider in the rational design of promising therapeutic candidates.

Molecular dynamics (MD) simulations provided critical insights that complemented both molecular docking data and MM/GBSA binding free energy estimations, offering a more comprehensive understanding of the behavior and stability of the ligand–receptor complexes over time. Among the analyzed compounds, ZINC34176694 and ZINC03876158 demonstrated notable hydrophobic and van der Waals interactions throughout the simulation trajectories. These non-covalent forces were particularly persistent and spatially well-distributed within the binding pocket, suggesting their fundamental contribution to the conformational stability of the protein–ligand complexes. The dynamic nature of these interactions reinforces their importance in maintaining the ligand in an energetically favorable binding mode during the simulation period.

Conversely, compounds such as ZINC03977981 and ZINC03876158 exhibited significant electrostatic interactions, which played a key role in the initial anchoring of the ligand to the receptor and in stabilizing the complex throughout the simulation. These electrostatic contributions, including salt bridges and long-range polar interactions, were consistently observed and are indicative of strong complementarity between the physicochemical properties of the ligands and the residues lining the active site of the androgen receptor.

In addition, the detailed analysis of specific interactions with functionally relevant amino acid residues—particularly Asn705, Arg752, and Met780—highlighted their critical role in ligand recognition and stabilization. These residues appear to act as anchoring points or “hot spots” within the binding cavity, mediating key hydrogen bonds and hydrophobic contacts that reinforce the binding affinity and specificity of the studied compounds. The recurrent engagement of these residues across different ligands suggests a conserved interaction profile that could be exploited in future rational design strategies.

Overall, the persistence and strength of these interactions over the course of the MD simulations support the hypothesis that the studied compounds, especially ZINC03876158, are promising candidates for further investigation and potential optimization as selective androgen receptor modulators (SARMs). These findings provide a solid structural and energetic foundation for guiding subsequent in silico modifications, SAR studies, and experimental validation.

### 2.10. MM/GBSA Binding Free Energy

To better understand the mode of interaction of these new inhibitors, it is important to estimate their binding affinities (ΔG_bind_) and the values of the other energy contributions. Binding free energy estimations using the MM/GBSA (Molecular Mechanics Generalized Born Surface Area) method serve as a reliable hybrid approach for quantifying protein–ligand interaction strength. This method integrates molecular mechanics with implicit solvation models, enabling the decomposition of binding energy into individual energetic contributions—van der Waals, electrostatic, polar, and non-polar solvation terms—thus providing a comprehensive thermodynamic profile of ligand affinity.

The ΔG_bind_ and the values of the energy contributions of van der Waals (ΔE_vdW_), electrostatic (ΔE_ele_), polar (ΔG_GB_), and non-polar (ΔG_nonpol_) forces are summarized in Table 8 (all results in Kcal/mol). As shown in Table 8, the binding free energy (ΔG_bind_) results agree with those from docking and MD simulations. ZINC34176694 emerged as the top candidate, exhibiting the strongest binding affinity (−56.42 kcal/mol), outperforming the reference compound CPA (−54.08 kcal/mol). This result was primarily driven by an enhanced van der Waals contribution (ΔEvdW = −61.75 kcal/mol), indicating a favorable fit within the hydrophobic environment of the AR binding site. This classification is in accordance with the values from the molecular docking, which suggests the same classification for these compounds.

ZINC03977981 also demonstrated promising thermodynamic behavior, with a ΔG_bind of −53.51 kcal/mol and a notably high electrostatic component (ΔEele = −30.28 kcal/mol), which exceeded that of the reference compound. This suggests that electrostatic interactions play a more dominant role in its binding stability, possibly offering enhanced specificity.

ZINC03876158 showed a competitive binding profile (ΔGbind = −52.31 kcal/mol), supported by strong electrostatic contributions and favorable hydrogen bonding with Met780, consistent with MD results.

Although ZINC04097308 exhibited the lowest overall ΔGbind (−51.83 kcal/mol), its balanced energy distribution and stable MD performance suggest sufficient binding site occupancy and structural compatibility for further investigation.

Hydrophobic interactions with Leu701, Leu704, Met780, and Phe876 were consistently observed across all compounds, reaffirming the critical role of these residues in binding site stabilization. This trend aligns with previous studies and underscores the importance of targeting hydrophobic hotspots for high-affinity ligand design.

The concordance among MM/GBSA, docking, and MD findings reinforces the robustness of this computational pipeline. In particular, ZINC34176694 stands out as a highly promising scaffold for further structure-based optimization and preclinical evaluation, with strong potential as a selective AR modulator.

### 2.11. Protein–Inhibitor Binding Affinity

**Figure 10:**
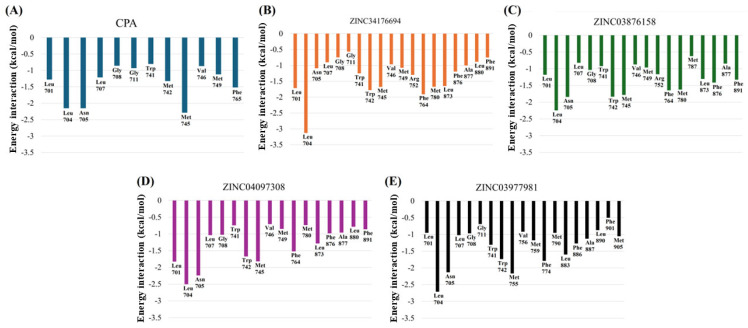
(**A**–**E**) Per-residue binding free energy decomposition analysis.

The decomposition analysis of the free energy of binding per residue, shown in Figure 10, provides a refined view of the molecular interactions that contribute most to the stability of ligand-receptor complexes. This approach makes it possible to identify the key residues responsible for anchoring compounds to the active site of the androgen receptor (AR), providing crucial input for the rational optimization of drugs.

It can be seen that residues Leu701, Leu704, Met780, Phe876, and Arg752 play a central role in stabilizing interactions, with markedly favorable energetic contributions. These residues, already well established in the literature as hotspots of the AR binding domain, showed significant negative ΔG values per interaction, indicating the predominant participation of hydrophobic interactions and hydrogen bonds, especially in the cases of Arg752 and Met780.

Among the compounds studied, ZINC34176694 stood out by showing a particularly intense energy contribution associated with the Arg752 residue, consistent with the pattern observed in the reference compound (CPA). This structural and functional similarity reinforces the potential of this ligand as a selective modulator of AR. In addition, the presence of π-alkyl and van der Waals interactions with Phe876 and Leu704 corroborates its high and stable affinity with the binding site, a fact also evidenced in the RMDS and MM/GBSA results.

The ZINC03876158 ligand exhibited prominent interactions with the residues Met780 and Leu701, characterized by a distinct electrostatic contribution. These interactions suggest a deeper and more specific anchoring within the receptor’s hydrophobic core, which may account for the high binding affinity observed, even in the presence of a relatively elevated average RMSD. The persistence of these contacts throughout the simulation indicates a stable and energetically favorable binding conformation, combining flexibility with strong site recognition.

Similarly, the compounds ZINC04097308 and ZINC03977981 demonstrated comparable energy distribution patterns, engaging the same critical residues, albeit with minor variations in interaction strength. This convergence reinforces the structural consistency of the androgen receptor’s binding site and highlights the relevance of Met780, Leu701, and Asn705 as key residues in ligand stabilization. These findings point to a conserved interaction profile across diverse ligands, providing a valuable reference for future design and optimization efforts.

## 3. Materials and Methods

### 3.1. Reference Compound Selection

Based on its crystallographic structure, the bioactive conformation of the ligand cyproterone acetate was determined by Bohl et al. [20]. This conformation was then used as the basis for the study of virtual screening by structural and electrostatic similarities in the ROCS and EON software [26].

### 3.2. Application of Rapid Overlay of Chemical Structures (ROCS) and Electrostatic Similarity (EON)

The Princeton and Zinc Drug databases were used to select molecules by virtual screening, in which the shape was approximated by superimposing Gaussian curves on the central atom and used to calculate the maximum volume crossing of two molecules. In this study, the algorithm (Gaussian functions) implemented in the ROCS software (http://www.eyesopen.com/rocs; accessed on 6 September 2023) was used to generate and evaluate the three-dimensional overlays of the database with the reference structure/model of cyproterone acetate to obtain 500 well-classified structures per database [57].

In order to determine whether the aligned chemicals from ROCS and EON (https://www.eyesopen.com/eon; accessed on 10 October 2023) had identical electrostatic potentials, additional analysis was conducted. The hit compounds were identified using the ET_Comb score, which combines the ShapeTanimoto (ST) and PB Electrostatic Tanimoto (ET_pb) scores [58].

### 3.3. In Silico Study of Pharmacokinetic and Toxicological Properties

The pharmacokinetic features, or ADME (absorption, distribution, metabolism, and excretion), were predicted in silico using the Biovia Discovery Studio Visualizer program (https://discover.3ds.com/discovery-studio-visualizer-download; accessed on 15 December 2024). The following descriptors were used by the software to evaluate the predictions: absorption in the human stomach, water solubility, penetration of the blood-brain barrier, binding to plasma proteins, CYP2D6 binding, and CYP3A4 binding.

Following the guidelines of Ramos et al. [59], the toxicity assessment was carried out using the DEREK software (version 10.0.2) (Deductive Estimation of Risk from Existing Knowledge). The software recognizes known toxicophoric groups in a chemical on the basis of warning structures.

The lethal dose (LD_50_ = mg/kg) and toxicity class characteristics used in this study were predicted from the Protox II online server (version 4.0) (http://tox.charite.de/protox_II/index.php?site=home; accessed on 2 January 2024) [36]. The predictions are based on the similarities of the functional groups of the investigated structures with data from structures that have already been validated in vitro and in vivo.

### 3.4. Molecular Docking Simulation

For the molecular docking study, we used the protein structure referring to the crystal structure of the binding domain to the mutant ligand of the human androgen receptor T877A with complexed cyproterone acetate (CPA), obtained from the Protein Data Bank (https://www.rcsb.org/; accessed on 15 August 2023) with the PDB code: 2OZ7 and 1.8 Å resolution for the Homo sapiens organism, which is the reference ligand [20]. Water molecules and other residues that can obstruct the ligand-macromolecule interaction were eliminated using the Biovia Discovery Studio Visualizer program (https://discover.3ds.com/discovery-studio-visualizer-download; accessed on 22 January 2024) [60].

The DockThor server (https://www.dockthor.lncc.br/v2/; accessed on 19 February 2024) was used to perform the molecular docking simulations [61]. Based on research published in the literature, the x, y, and z coordinates of the target’s (Androgen Receptor, or AR) active site (see Table 9) correspond to the interaction between AR and its standard ligand, CPA [57]. The program performs conventional docking, using a genetic algorithm as the search function and the MMFF94 force field as the evaluation function, with a Grid docking approach.

The Grid size was standardized at 20 × 20 × 20 Å and 0.25 discretization. The accuracy of the algorithm was determined by the following values: Number of evaluations (*n* = 1,000,000), population size (*n* = 750), number of runs (*n* = 24) in order to analyze the conformations, the interactions of the molecules with amino acid residues of the protein and the binding energy [61].

The molecular docking protocol was validated through the redocking process to see if the software is capable of correctly predicting the position of the ligand in the protein’s active site [62]. In order to determine the RMSD (root-mean-square-deviation) values of the crystallographic pose of the ligand in relation to the computational pose, in this case the crystallographic ligand itself was subjected to the molecular docking method. The RMSD value between the lowest energy poses, which are the conformations acquired by the molecule, can be used as a validation method when compared with the existing crystallographic model pose [63,64].

The similarity calculation was based on the comparison of the amount of amino acid residues present in the reference molecule that interacted with the androgen receptor with the amino acid residues present in the selected ligands.

### 3.5. Synthetic Accessibility

Synthetic accessibility prediction (SA) was performed for the best results obtained in molecular docking through the AMBIT-SA software and the SwissADME online server.

AMBIT-SA Version 3.1.0 (ambit.sourceforge.net/reactor.html; accessed on 9 March 2024) [65] is a stereochemistry-based server that analyzes the topological and structural properties of molecules under investigation. The algorithm works with a score between 0 and 100, with a score of 100 representing an easily synthesizable molecule [66]. The SwissADME server (http://www.swissadme.ch/; accessed on 11 March 2024), SA parameters vary between 1 (easily synthesized molecules) and 10 (difficultly synthesized molecules).

### 3.6. In Silico Prediction of Molecular Properties

The molecular properties of the compounds selected by molecular docking were predicted by the Molinspiration (version 2021.10) (https://www.molinspiration.com/cgi/properties; accessed on 20 March 2024) and SwissADME software. For the calculation of important molecular parameters, including logP, polar surface area, number of hydrogen bond donors and acceptors and others, Molinspiration offers free web services [67]. The SwissADME software (http://www.swissadme.ch/; accessed on 11 March 2024) allowed the interpretation of the Bioavailability Radar graph, which correlates data such as molecule size, flexibility, solubility, lipophilicity, saturation, and polarity, which also makes it possible to predict whether the analyzed molecules will have good oral bioavailability.

### 3.7. In Silico Determination of Biological Activity

The Prediction of Activity Spectral for Substances (PASS) web server (http://www.pharmaexpert.ru/passonline/index.php; accessed on 19 March 2024) was used to evaluate the biological activity of the selected substances. It makes instant predictions about a wide range of biological activity based on molecular structure. The variables Pa (probable activity) and Pi (probable inactivity) are used to calculate the activity. The two factors that influence the prediction of PASS are Pa and Pi, whose values are between 0 and 1. Therefore, the Pa and Pi values of ligands can be used to predict whether they are potentially active or inactive [46].

### 3.8. Prediction of Lipophilicity and Water Solubility

According to the methodology proposed by Sepay et al. [68], the values for lipophilicity and solubility were determined using the SwissADME server (http://www.swissadme.ch/; accessed on 19 March 2024), analyzing the iLOGP, XLOGP, WLOGP, MLOGP, and SILI-COS-IT methods for lipophilicity and the ESOL, ALI and SILICOS-IT methods for solubility in water. With the extensive database on this website, you can make very accurate guesses about the physicochemical properties, lipophilicity, water solubility, pharmacokinetics, drug likeness, and medicinal chemistry properties.

### 3.9. Molecular Dynamics Study

The molecular atomic charges were obtained with the Restrained Electrostatic Potential (RESP) protocol using the Hartree-Fock method with the 6-31G* base set [69]. The amino acid protonation state was characterized in neutral pH using the PDB2PQR server [70]. The parameters for each molecule were constructed using the General Amber Force Field (GAFF) [71]. Amber 16 packages were used for the MD simulations [72]. The ff14SB force field [73] was used for all MD simulations. The absent hydrogens in the protein crystal were added by the tLEaP module during the process of building the complexes. The systems were solvated in an octahedron periodic box containing explicit water molecules described by the TIP3P model [74]. The distance chosen for the shear radius was 12 Å for all directions of the solvent from the solute.

The Particle Mesh Ewald method was used for the calculation of electrostatic interactions [75], and bonds involving hydrogen atoms were restricted with the SHAKE algorithm [76]. The simulation of MD was divided into stages of energy minimization, heating, equilibrium, and production. The sander module was used for both steps of energy minimization, where the steepest descent method and conjugate gradient algorithm were employed to perform 1500 cycles divided among the steps. In the first step, the solute was restricted with a constant harmonic force of 100 kcal/mol.Å^−2^, while the water and anti-ion molecules were free. In the second stage, the complexes were totally free to move.

Then, the systems were gradually heated for 600 ps until the temperature reached 300 K. The heating was divided into five stages, where the collision frequency was 3.0 ps^−1^ and the Langevin thermostat was used for temperature control [77]. The heavy atoms were restricted with a constant harmonic force of 50 kcal/mol.Å^−2^ during the initial four steps. In the last heating step, the constant harmonic force was removed. These simulations were performed at constant volume (NVT). In the equilibrium stage, the systems were submitted to a simulation of 5 nanoseconds (ns) with a temperature of 300 K and constant pressure. During the production stage, 100 ns of MD simulations were generated.

### 3.10. Free Energy Calculations Using the MM/GBSA Approach and Protein–Inhibitor Binding Affinity

The free energy of each complex was obtained from the last 5 ns of the trajectory, corresponding to 500 snapshots. In the MM-GBSA approach, binding free energy is calculated from the free energy of a linker interacting with a receptor to form the complex [78].

The equations that describe the calculations of the energy are:

ΔG_bind_ = ΔH − TΔ ≈ ΔE_MM_ + ΔG_sol_ + TΔS, (1)

ΔEMM = ΔE_internal_ + EΔ_electrostatic_ + ΔE_vdw_ (2)

ΔG_sol_ = ΔG_GB_ + ΔG_SASA_ (3)

As shown in Equation (1), the enthalpy part is expressed as the summation of the molecular mechanical energy (ΔE_MM_) and the solvation free energy (ΔG_sol_), where ΔE_MM_ is composed of the intra-molecular energy (ΔE_internal_, including the bond, angle, and dihedral energies of the system), the electrostatic energy (ΔE_ele_) and the van der Waals interactions (ΔE_vdW_) (Equation (2)). The solvation free energy (ΔG_sol_) is also composed of two parts, namely, the polar part (ΔG_pol_) and the non-polar (ΔG_np_) part (Equation (3)), where ΔG_pol_ is usually computed by the Generalized Born (GB) model, while ΔG_np_ is estimated by the solvent accessible surface area (SASA)-based approach [79].

In addition to the total binding free energy, a per-residue energy decomposition analysis was performed to identify key residues contributing to complex stabilization and ligand affinity. These calculations enabled the characterization of critical interaction hotspots within the receptor binding site, supporting structural insights obtained from molecular docking and MD simulations. All calculations were carried out using the Amber 16 package.

## 4. Conclusions

This study identified potential candidates for prostate cancer inhibitors through the application of chemoinformatics tools. Docking and molecular dynamics simulations demonstrated the interaction and stability of the selected compounds (ZINC34176694, ZINC03876158, ZINC04097308, and ZINC03977981) in the active site of the androgen receptor. In particular, ZINC34176694 stands out as a highly promising scaffold for structural optimization and preclinical evaluation, with strong potential as a selective AR modulator. The compounds also presented adequate results within the pharmacokinetic and toxicological parameters, showing themselves to be promising candidates for the development of new drugs.

## Figures and Tables

**Table 1:** Computational pharmacokinetic parameters (ADME) from the Princeton and Zinc Drug Database databases of the selected molecules.

Molecules	ADME					
	CYP2D6 ^[a]^	CYP3A4 ^[b]^	Solubility ^[c]^	HIA ^[d]^	PPB ^[e]^	BBB ^[f]^
CPA	false	false	2	0	false	2
PRINCETON_01	false	false	3	0	false	3
PRINCETON_02	false	false	2	0	false	3
PRINCETON_03	false	false	3	0	false	3
PRINCETON_04	false	false	3	0	false	3
PRINCETON_05	false	false	3	0	false	3
ZINC04097308	false	false	3	0	false	3
ZINC03830600	false	false	3	0	false	3
ZINC03977981	false	false	3	0	false	3
ZINC04212851	false	false	3	0	false	3
ZINC13540519	false	false	3	0	false	3
ZINC36388590	false	true	2	0	false	2
ZINC03875357	false	false	3	0	false	3
ZINC03876158	false	false	3	0	false	3
ZINC04097304	false	false	3	0	false	3
ZINC04212854	false	false	3	0	false	3
ZINC34176695	false	true	2	0	false	2
ZINC03833821	false	false	3	0	false	3
ZINC03830602	false	false	3	0	false	3
ZINC34176694	false	true	2	0	false	2
ZINC04340274	false	false	3	0	false	3
ZINC03831269	false	false	3	0	false	3
ZINC03831270	false	false	3	0	false	3
ZINC03876136	false	false	3	0	false	3
ZINC03830599	false	false	3	0	false	3

^[a]^ Inhibition of cytochrome P450 (CYP450) 2D6 (false—Non-inhibitory; true—Inhibitory). ^[b]^ Inhibition of cytochrome P450 (CYP450) 2D6 (false—Non-inhibitory; true—Inhibitory). ^[c]^ Aqueous solubility (acceptable range: range 0–3) where 3 is good solubility. ^[d]^ HIA—human intestinal absorption (acceptable range: range is 0–2, where 0 is good absorption). ^[e]^ PPB—Binding to plasma proteins (false—does not bind to plasma proteins; true—binds to plasma proteins). ^[f]^ BBB—blood-brain barrier (0—Very high penetrant; 1 (high); 2 (medium); 3 (low); 4 (very low).

**Table 2:** Binding affinity and interactions of promising molecules with the androgen receptor.

Molecules	∆G (kcal mol^−1^)	Amino Acids That Interact by Hydrogen Bonding	Amino Acids That Form Hydrophobic Interactions
CPA (Reference)	−10.827	Asn705, Arg752	Ala877, Gln711, Gly708, Ile899, Leu701, Leu704, Leu707, Leu873, Leu880, Met742, Met745, Met749, Met780, Met787, Met895, Phe697, Phe764, Phe876, Phe891, Ser778, Trp741, Val746.
ZINC34176694	−10.850	Arg752	Ala877, Arg779, Asn705, Gln711, Gly708, Ile899, Leu701, Leu704, Leu707, Leu873, Leu880, Met742, Met745, Met749, Met780, Phe697, Phe764, Phe876, Ser778, Trp741, Val746.
ZINC03876158	−10.369	Met780	Ala877, Arg752, Asn705, Gln711, Gly708, Ile899, Leu701, Leu704, Leu707, Leu873, Leu880, Met742, Met745, Met749, Met787, Met895, Phe764, Phe876, Phe891, Trp741, Val746.
ZINC04097308	−10.260	Ala877, Arg752, Asn705	Gln711, Gly708, Ile899, Leu701, Leu704, Leu707, Leu873, Leu880, Leu881, Met742, Met745, Met749, Met780, Met787, Met895, Phe697, Phe764, Phe876, Phe891, Ser778, Trp741, Val746.
ZINC03977981	−10.237	Asn705	Ala877, Arg752, Gln711, Gly708, Ile899, Leu701, Leu704, Leu707, Leu873, Leu880, Leu881, Met742, Met745, Met749, Met780, Met787, Met895, Phe697, Phe764, Phe876, Phe891, Ser778, Trp741, Val746.

**Table 3:** Synthetic accessibility prediction using the free web tools AMBIT and SwissADME.

Molecules	AMBIT-SA ^[a]^	SwissADME ^[b]^
	SA SCORE *	
CPA (Reference)	23.712	5.54
ZINC34176694	23.097	5.82
ZINC03876158	31.245	5.40
ZINC04097308	20.114	5.91
ZINC03977981	22.738	6.02

* Synthetic accessibility (SA); ^[a]^ there are three different accessibility scores for the AMBIT-SA web server: easy (score ≥ 50), medium (10 < score ≤ 49), and difficult (score ≤ 10); ^[b]^ the range of SwissADME: scores is 1 (very easy) to 10 (extremely difficult).

**Table 4:** Pharmacokinetic parameters for good oral availability of compounds.

Molecules	Molinspirations Calculations							
	Vol ^[a]^	TPSA ^[b]^	NROTB ^[c]^	HBA ^[d]^	HBD ^[e]^	LogP ^[f]^	MW ^[g]^	Lipinski’s Violations
Rule	-	-	-	≤10	≤5	≤5	≤500	≤1
CPA (Reference)	376.63	60.45	3	4	0	4.44	416.94	0
ZINC34176694	421.86	80.67	5	5	1	3.69	484.97	0
ZINC03876158	349.81	74.60	1	4	2	2.39	376.47	0
ZINC04097308	396.78	93.07	2	6	2	2.61	436.52	0
ZINC03977981	395.20	93.07	2	6	2	2.57	452.49	0

^[a]^ Vol.—Molecular volume; ^[b]^ TPSA—Topological Polar Surface Area; ^[c]^ NROTB—Number of Rotating Connections; ^[d]^ HBA—Number of Hydrogen Bond Donors; ^[e]^ HBD—Number of Hydrogen Bond Acceptors; ^[f]^ LogP—Partition Coefficient; ^[g]^ MW—Molecular Weight.

**Table 5:** Biological activity prediction of compounds from virtual screening.

Molecules	Biological Activity	Pa ^[a]^	Pi ^[b]^
CPA (Reference)	Androgen antagonist	0.900	0.002
	Prostate disorders treatment	0.736	0.006
	Antineoplastic	0.742	0.019
	Prostatic (benign) hyperplasia treatment	0.624	0.004
	AR expression inhibitor	0.485	0.029
	Prostate cancer treatment	0.402	0.021
ZINC34176694	Androgen antagonist	0.710	0.003
	Antineoplastic (non-Hodgkin’s lymphoma)	0.655	0.008
	Prostate disorders treatment	0.327	0.062
ZINC03876158	Androgen antagonist	0.993	0.002
	Antineoplastic (non-Hodgkin’s lymphoma)	0.647	0.008
	AR expression inhibitor	0.599	0.013
	Prostate disorders treatment	0.523	0.019
ZINC04097308	Prostate disorders treatment	0.485	0.023
	Androgen agonist	0.450	0.004
	Antineoplastic	0.484	0.077
	Antimetastatic	0.440	0.035
	Antineoplastic (non-Hodgkin’s lymphoma)	0.427	0.084
	Prostatic (benign) hyperplasia treatment	0.365	0.013
	Anticarcinogenic	0.362	0.038
ZINC03977981	Antineoplastic (non-Hodgkin’s lymphoma)	0.648	0.008
	Antineoplastic (multiple myeloma)	0.391	0.026
	Antimetastatic	0.363	0.058
	Androgen antagonist	0.150	0.014
	Prostate disorders treatment	0.238	0.118
	Prostatic (benign) hyperplasia treatment	0.113	0.061

^[a]^ Pa = Probability to be active; ^[b]^ Pi = Probability to be inactive.

**Table 6:** Lipophilicity prediction via the SwissADME online server.

Molecules	iLOGP	XLOGP	WLOGP	MLOGP	SILICOS-IT	Consensus LogP
CPA (Reference)	3.41	3.64	4.61	3.71	4.51	3.98
ZINC34176694	2.63	3.75	4.89	3.28	4.63	3.84
ZINC03876158	1.48	2.00	3.34	2.45	3.19	2.49
ZINC04097308	3.15	1.38	2.92	1.73	3.03	2.44
ZINC03977981	2.69	2.48	3.21	1.74	3.03	2.63

**Table 8:** Affinity energy values (values in kcal/mol). ΔE_vdW_, contributions by van der Waals interactions; ΔE_ele_, electrostatic energy; ΔG_GB_, polar solvation energy; ΔG_nonpol_, non-polar solvation energy; ΔG_MMGBSA_, binding free energy estimations using the MM/GBSA (Molecular Mechanics Generalized Born Surface Area) method.

Molecules	ΔE_vdW_	ΔE_ele_	ΔG_GB_	ΔG_nonpol_	ΔG_MMGBSA_
CPA	−57.89	−10.74	21.57	−7.01	−54.08
ZINC34176694	−61.75	−21.79	35.03	−7.91	−56.42
ZINC03876158	−53.85	−27.30	35.64	−6.80	−52.31
ZINC04097308	−58.39	−16.43	30.60	−7.61	−51.83
ZINC03977981	−57.24	−30.28	41.54	−7.52	−53.51

**Table 7:** Water solubility ^*^ prediction using the SwissADME online server.

Molecules *	ESOL	Ali	SILICOS-TI	Consensus LogS
CPA (Reference)	−4.52 (ms)	−4.60 (ms)	−4.78 (ms)	−4.63 (ms)
ZINC34176694	−4.88 (ms)	−5.14 (ms)	−5.00 (ms)	−5.01 (ms)
ZINC03876158	−3.37 (s)	−3.19 (s)	−3.37 (s)	−3.31 (s)
ZINC04097308	−3.28 (s)	−2.94 (s)	−3.48 (s)	−3.23 (s)
ZINC03977981	−4.08 (ms)	−4.08 (ms)	−3.49 (s)	−3.88 (s)

* Solubility (s = Soluble, ms = Moderately soluble, ps = Slightly soluble).

**Table 9:** Coordinates of the molecular target active site.

Receptor	Ligand	Coordinates of Grid Center	Grid Box Size
Androgen Receptor (*Homo sapiens*) (PDB ID: 2OZ7)	6-chloro-1β,2βα-dihydro-17-hydroxy-3′H-cyclopropa[1,2] pregna-1,4,6-triene-3,20-dione acetate	X = 26.9229 Y = 1.3605 Z = 2.9661	20x 20y 20z

## Data Availability

Data Availability in this study is available in this article or upon request from the corresponding author.

## Supplementary Materials

The following supporting information can be downloaded at: https://www.mdpi.com/article/10.3390/ph18060888/s1, Table S1. Plasma protein binding prediction. Table S2. Predictions of toxicological properties and LD_50_. Table S3. Result of binding affinity values from the molecular docking process. Figure S1. Interaction of the pivot molecule with AR, interaction diagram (a) 2D and (b) 3D. Figure S2. Interaction of the molecule ZINC34176694 with AR, interaction diagram (a) 2D and (b) 3D. Figure S3. Interaction of the molecule ZINC03876158 with AR, interaction diagram (a) 2D and (b) 3D. Figure S4. Interaction of the molecule ZINC04097308 with AR, interaction diagram (a) 2D and (b) 3D. Figure S5. Interaction of the molecule ZINC03977981 with AR, interaction diagram (a) 2D and (b) 3D. Figure S6. Bioavailability radar graph. The ideal physicochemical region for oral bioavailability is the colored zone. Figure S7. The bioavailability radar for the selected compounds and the reference molecule. Figure S8. Predicted lipophilicity values (LogPo/w) of the promising compounds and the pivot compound. Figure S9. Predicted water solubility values (LogS) of the lead compounds and the pivot molecule.
