# Supplementary material for: Identification of Inhibitors with Potential Anti-Prostate Cancer Activity: A Chemoinformatics Approach
Source: Pharmaceuticals (Basel). 2025 Jun 13;18(6):888. doi: 10.3390/ph18060888 (PMC12196096; doi:10.3390/ph18060888)

pharmaceuticals

MDPI

Article

Identification of inhibitors with potential anti-prostate cancer activity: a chemoinformatics approach

Norberto S. Costa[1,2]; Lúcio R. Lima[2]; Jorddy N. Cruz[2], Igor V. F. Santos[3]; Rai C. Silva[2]; Alexandre A. Maciel[2]; Elcimar S. Barros[2]; Ryan S. Ramos[2,3]; Njogu M. Kimani[4]; Alberto Aragón-Muriel[5]; Juan M. Álvarez-Caballero[6]; Joaquín M. Campos [7,8] and Cleydson B.R. Santos [1,2,3*]

1 Graduate Program in Pharmaceutical Sciences at the Federal University of Amapá, Macapá 68902-280, AP, Brazil; nscquimica@gmail.com;
2 Laboratory of Modeling and Computational Chemistry, Department of Biological and Health Sciences, Federal University of Amapá, Macapá 68902-280, AP, Brazil; luciorolima@gmail.com; jorddynevescruz@gmail.com; raics@usp.br; alexandremaciell@yahoo.com.br; barrositb2008@hotmail.com; ryanquimico@gmail.com
3 Graduate Program in Biotechnology and Biodiversity-Network BIONORTE, Federal University of Amapá, Macapá 68902-280, AP, Brazil; igorsantosvictor@gmail.com
4 Natural Product Chemistry and Computational Drug Discovery Laboratory, University of Embu, P.O. Box 6-60100, Embu, Kenya; njogu.mark@embuni.ac.ke
5 Grupo de Investigaciones Bioquímicas, Universidad del Magdalena, CP 470004, Santa Marta, Colombia; aaragonm@unimagdalena.edu.co
6 Grupo de Química y Bioprospección de Productos Naturales, Departamento de Química, Universidad del Magdalena, CP 470004, Santa Marta, Colombia; jalvarez@unimagdalena.edu.com
7 Department of Pharmaceutical and Organic Chemistry, Faculty of Pharmacy, Campus of Cartuja, University of Granada, 18071 Granada, Spain
8 Biosanitary Institute of Granada (ibs.GRANADA), University of Granada, 18071 Granada, Spain

* Correspondence: breno@unifap.br; Tel.: Tel.: +55-(96)-4009-2699

SUPPLEMENTARY MATERIAL

Table S1. Plasma protein binding prediction.

Table S2. Predictions of toxicological properties and LD_{50}.

Table S3. Result of binding affinity values from the molecular docking process.

Figure S1. Interaction of the pivot molecule with AR, interaction diagram (a) 2D and (b) 3D.

Figure S2. Interaction of the molecule ZINC34176694 with AR, interaction diagram **(a)** 2D and **(b)** 3D.

Figure S3. Interaction of the molecule ZINC03876158 with AR, interaction diagram **(a)** 2D and **(b)** 3D.

Figure S4. Interaction of the molecule ZINC04097308 with AR, interaction diagram **(a)** 2D and **(b)** 3D.

Figure S5. Interaction of the molecule ZINC03977981 with AR, interaction diagram **(a)** 2D and **(b)** 3D.

Figure S6. Bioavailability radar graph. The ideal physicochemical region for oral bioavailability is the colored zone. LIPO (lipophilicity): $-0.7 < XLOGP3 < +5.0$; SIZE: 150 g/mol < MW < 500 g/mol; POLAR (polarity): 20 Å^2 < TPSA < 130 Å^2; INSOLU (insolubility): −6 < LogS (ESOL) < 0; INSATU (in- saturation): 0.25 < Fraction Csp3 < 1; FLEX (flexibility): 0 < number. rotatable bonds < 9 [44].

Figure S7. The bioavailability radar for the selected compounds and the reference molecule

Figure S8. Predicted water solubility values (LogS) of the lead compounds and the pivot molecule.

Figure S9. Predicted water solubility values (LogS) of the lead compounds and the pivot molecule.

Table S1. Plasma protein binding prediction.

**Molecules**	**ADME**	
	**PPB**	PPB_Applicability_Value[(a)]
CPA	false	0,0168724
PRINCETON_338556	false	0,927296
PRINCETON_549113	false	0,755386
PRINCETON_209731	false	0,789942
PRINCETON_417096	false	0,847778
PRINCETON_305985	false	0,076437
ZINC04097308	false	0,995619
ZINC03830600	false	0,906129
ZINC03977981	false	0,999465
ZINC04212851	false	0,999879
ZINC13540519	false	0,99923
ZINC36388590	false	0,61432
ZINC03875357	false	0,993774
ZINC03876158	false	0,95337
ZINC04097304	false	0,999249
ZINC04212854	false	0,999898
ZINC34176695	false	0,61432
ZINC03833821	false	0,999986
ZINC03830602	false	0,906129
ZINC34176694	false	0,61432
ZINC04340274	false	0,906129
ZINC03831269	false	0,999878
ZINC03831270	false	0,999878
ZINC03876136	false	0,999989
ZINC03830599	false	0,906129

(a) The closer the value is to zero, the greater the possibility of binding to plasma proteins.

Table S2. Predictions of toxicological properties and LD_{50}.

Compounds	Toxicity Prediction Alert (Lhasa Prediction)	Toxicophoric Group	Toxicity alert	LD_{50} Toxic [1]	Toxicity class [2]
Pivô	Carcinogenicity in mouse and rat	Progestogen or derivative	PLAUSIBLE	5000	V
	Hepatotoxicity in human, mouse and rat	Halogenated hydrocarbon			
	Non-specific genotoxicity in vitro in human and mouse	3-Keto,4-delta,6-delta steroid	CERTAIN		
	Non-specific genotoxicity in vitro in mouse	3-Keto,4-delta,6-delta steroid	PROBABLE		
	Non-specific genotoxicity in vivo in human and mouse	3-Keto,4-delta,6-delta steroid	PROBABLE		
	Non-specific genotoxicity in vitro in rat	3-Keto,4-delta,6-delta steroid	CERTAIN		
PRINCETON-01	–	–	NO ALERTS	696	IV
PRINCETON-02	–	–	NO ALERTS	800	IV
PRINCETON-04	–	–	NO ALERTS	2000	IV
PRINCETON-05	–	–	NO ALERTS	500	IV
PRINCETON-10	–	–	NO ALERTS	602	IV
ZINC04097308_1_1	In vivo ocular toxicity in human, mouse and rat	Corticosteroid	PLAUSIBLE	1451	IV
	Skin sensitization in human, mouse and rat	1,2–Dicarbonyl or precursor	PLAUSIBLE		
	Teratogenicity in human, mouse and rat	Glucocorticoid	PLAUSIBLE		
ZINC03830600_1_3	In vivo ocular toxicity in human, mouse and rat	Corticosteroid	PLAUSIBLE	5010	VI
	Skin sensitization in human, mouse and rat	1,2–Dicarbonyl or precursor	PLAUSIBLE		
	Teratogenicity in human, mouse and rat	Glucocorticoid	PLAUSIBLE		
ZINC03977981_1_1	In vivo ocular toxicity in human, mouse and rat	Corticosteroid	PLAUSIBLE	4000	V
	Skin sensitization in human, mouse and rat	1,2–Dicarbonyl or precursor	PLAUSIBLE		
	Teratogenicity in human, mouse and rat	Glucocorticoid	PLAUSIBLE		
	In vivo ocular toxicity in	Corticosteroid	PLAUSIBLE	3710	V

ZINC04212851_1_1	human, mouse and rat				
	Skin sensitization in human, mouse and rat	1,2–Dicarbonyl or precursor	PLAUSIBLE		
	Teratogenicity in human, mouse and rat	Glucocorticoid	PLAUSIBLE		
ZINC13540519_1_1	In vivo ocular toxicity in human, mouse and rat	Corticosteroid	PLAUSIBLE	5000	V
	Skin sensitization in human	1,2–Dicarbonyl or precursor	CERTAIN		
	Skin sensitization in mouse and rat	1,2–Dicarbonyl or precursor	PROBABLE		
	Teratogenicity in human and rat	Glucocorticoid	PROBABLE		
	Teratogenicity in mouse	Glucocorticoid	CERTAIN		
ZINC36388590_1_3	Chromosome damage in vitro human, mouse and rat	Alpha-halo carbonyl compound	PLAUSIBLE	3000	V
	Eye irritation in human, mouse and rat	Alpha-Halo ketone	PLAUSIBLE		
	Lacrimation in human, mouse and rat	Alpha-Halo ketone	PLAUSIBLE		
	Skin sensitization in human, mouse and rat	Haloalkane	PLAUSIBLE		
ZINC03875357_1_1	In vivo ocular toxicity in human	Corticosteroid	CERTAIN	1680	IV
	In vivo ocular toxicity in mouse and rat	Corticosteroid	PROBABLE		
	Skin sensitization in human, mouse and rat	1,2–Dicarbonyl or precursor	PLAUSIBLE		
	Teratogenicity in human, mouse and rat	Glucocorticoid	PLAUSIBLE		
ZINC03876158_1_1	In vivo ocular toxicity in human, mouse and rat	Corticosteroid	PLAUSIBLE	3000	V
	Teratogenicity in human, mouse and rat	Glucocorticoid	PLAUSÍVEL		
ZINC04097304_1_1	In vivo ocular toxicity in human, mouse and rat	Corticosteroid	PLAUSIBLE	4500	V
	In vivo ocular toxicity in human, mouse and rat	Corticosteroid	PLAUSIBLE		
	Skin sensitization in human, mouse and rat	1,2–Dicarbonyl or precursor	PLAUSIBLE		
ZINC04212854_1_1	Teratogenicity in human, mouse and rat	Glucocorticoid	PLAUSIBLE	3000	V
	In vivo ocular toxicity in human, mouse and rat	Corticosteroid	PLAUSIBLE		

	Skin sensitization in human, mouse and rat	1,2–Dicarbonyl or precursor	PLAUSIBLE		
ZINC34176695_1_0	Chromosome damage in vitro human, mouse and rat	Alpha-halo carbonyl compound	PLAUSIBLE	3000	V
	Eye irritation in human, mouse and rat	Alpha-Halo ketone	PLAUSIBLE		
	Lacrimation in human, mouse and rat	Alpha-Halo ketone	PLAUSIBLE		
	Skin sensitization in human, mouse and rat	Haloalkane	PLAUSIBLE		
ZINC03833821_1_1	In vivo ocular toxicity in human and mouse	Corticosteroid	PLAUSIBLE	1680	IV
	In vivo ocular toxicity in human and rat	Corticosteroid	CERTAIN		
	Skin sensitization in human, mouse and rat	1,2–Dicarbonyl or precursor	PLAUSIBLE		
	Teratogenicity in human	Glucocorticoid	PLAUSIBLE		
	Teratogenicity in mouse and rat	Glucocorticoid	CERTAIN		
ZINC03830602_1_1	In vivo ocular toxicity in human, mouse and rat	Corticosteroid	PLAUSIBLE	5010	VI
	Skin sensitization in human, mouse and rat	1,2–Dicarbonyl or precursor	PLAUSIBLE		
	Teratogenicity in human, mouse and rat	Glucocorticoid	PLAUSIBLE		
ZINC34176694_1_3	Chromosome damage in vitro human, mouse and rat	Alpha-halo carbonyl compound	PLAUSIBLE	3000	V
	Eye irritation in human, mouse and rat	Alpha-Halo ketone	PLAUSIBLE		
	Lacrimation in human, mouse and rat	Alpha-Halo ketone	PLAUSIBLE		
	Skin sensitization in human, mouse and rat	Haloalkane	PLAUSIBLE		
ZINC04340274_1_1	In vivo ocular toxicity in human, mouse and rat	Corticosteroid	PLAUSIBLE	5010	VI
	Skin sensitization in human, mouse and rat	1,2–Dicarbonyl or precursor	PLAUSIBLE		
	Teratogenicity in human, mouse and rat	Glucocorticoid	PLAUSIBLE		
ZINC03831269_1_1	In vivo ocular toxicity in human, mouse and rat	Corticosteroid	PLAUSIBLE	4000	V
	Skin sensitization in human, mouse and rat	1,2–Dicarbonyl or precursor	PLAUSIBLE		
	Teratogenicity in	Glucocorticoid	PLAUSIBLE		

	human, mouse and rat				
ZINC03831270_1_0	In vivo ocular toxicity in human, mouse and rat	Corticosteroid	PLAUSIBLE	4000	V
	Skin sensitization in human, mouse and rat	1,2–Dicarbonyl or precursor	PLAUSIBLE		
	Teratogenicity in human, mouse and rat	Glucocorticoid	PLAUSIBLE		
ZINC03876136_1	In vivo ocular toxicity in human, mouse and rat	Corticosteroid	PLAUSIBLE	3000	V
	Skin sensitization in human, mouse and rat	1,2–Dicarbonyl or precursor	PLAUSIBLE		
	Teratogenicity in human, mouse and rat	Glucocorticoid	PLAUSIBLE		
ZINC03830599_1_1	In vivo ocular toxicity in human, mouse and rat	Corticosteroid	PLAUSIBLE	5010	VI
	Skin sensitization in human, mouse and rat	1,2–Dicarbonyl or precursor	PLAUSIBLE		
	Teratogenicity in human, mouse and rat	Glucocorticoid	PLAUSIBLE		

Table S3. Result of binding affinity values from the molecular docking process.

Compound	ΔG (kcal mol^{-1})	Percent similarity (amino acid residues)
**CPA**	**-10.827**	
ZINC34176694	-10.850	87.5%
ZINC34176695	-10.742	84.7%
ZINC03830620	-10.510	82.5%
ZINC36388590	-10.494	87.5%
ZINC03830618	-10.420	82.5%
ZINC03876158	-10.369	91.7%
ZINC03814363	-10.318	82.5%
ZINC04097308	-10.260	100.0%
ZINC03876136	-10.257	87.5%
ZINC03977981	-10.237	100.0%
PRINCETON-01	-10.107	87.5%
PRINCETON-02	-9.724	79.1%
PRINCETON-03	-9.712	75.0%
PRINCETON-04	-9.710	75.0%

Figure S1. Interaction of the pivot molecule with AR, interaction diagram (a) 2D and (b) 3D.

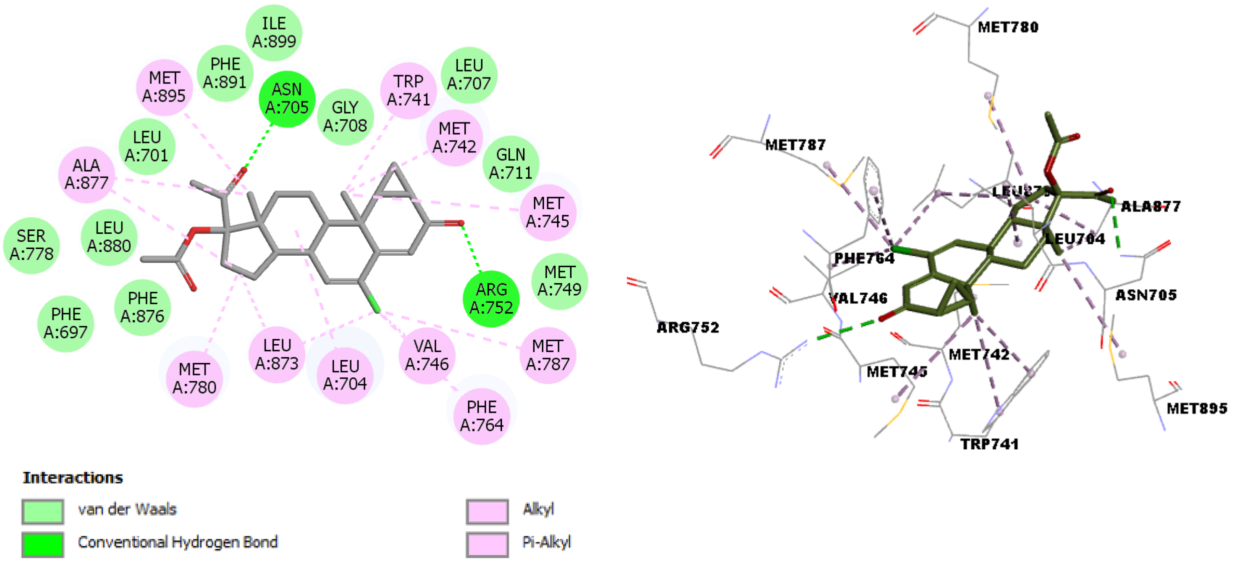

(**a**) (**b**)

Figure S2. Interaction of the molecule ZINC34176694 with AR, interaction diagram **(a)** 2D and **(b)** 3D.

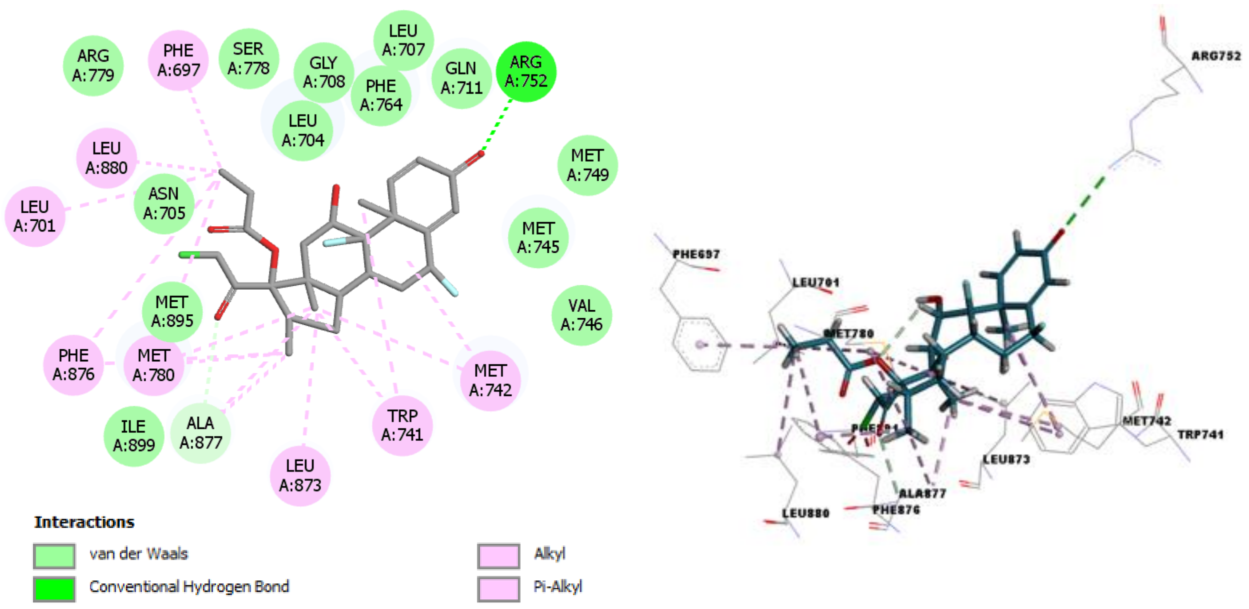

(**a**) (**b**)

Figure S3. Interaction of the molecule ZINC03876158 with AR, interaction diagram **(a)** 2D and **(b)** 3D.

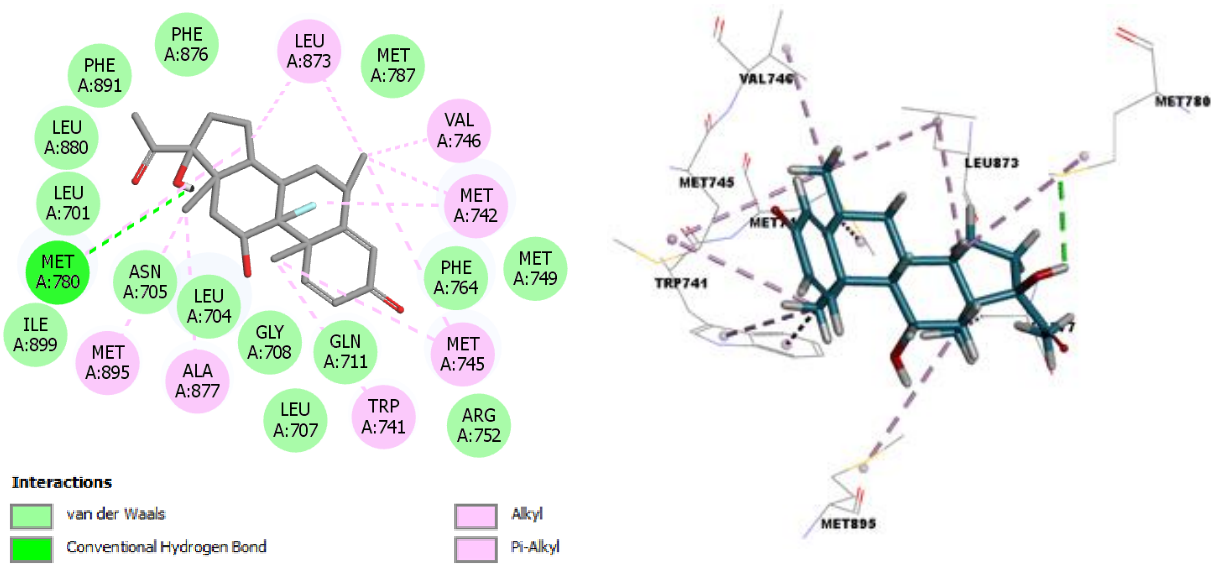

(**a**) (**b**)

Figure S4. Interaction of the molecule ZINC04097308 with AR, interaction diagram **(a)** 2D and **(b)** 3D.

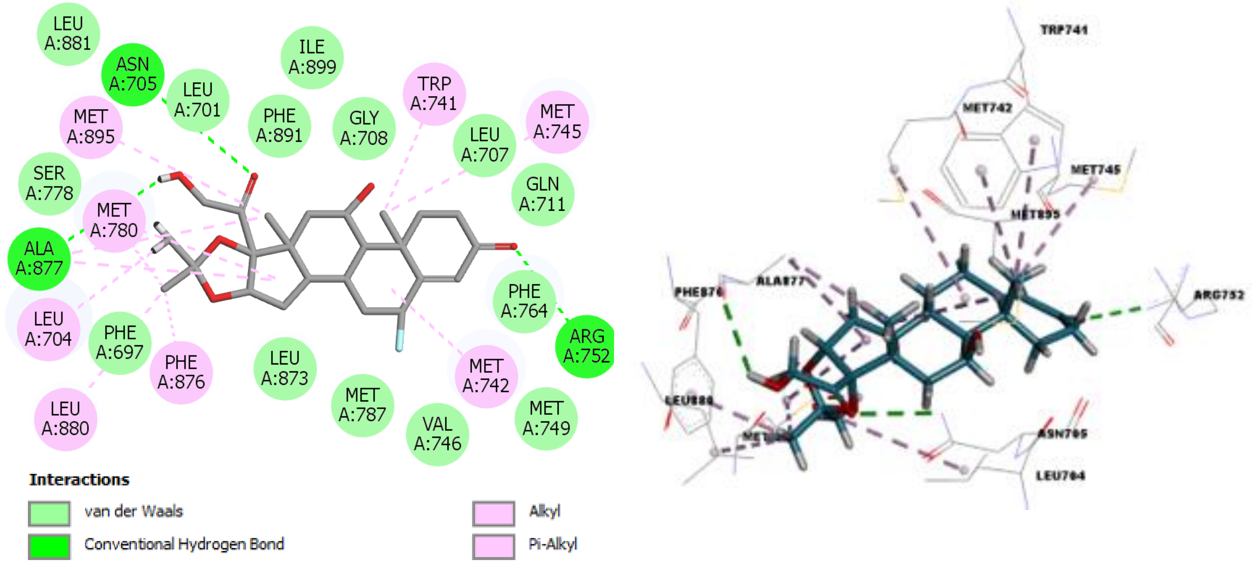

(**a**) (**b**)

Figure S5. Interaction of the molecule ZINC03977981 with AR, interaction diagram **(a)** 2D and **(b)** 3D.

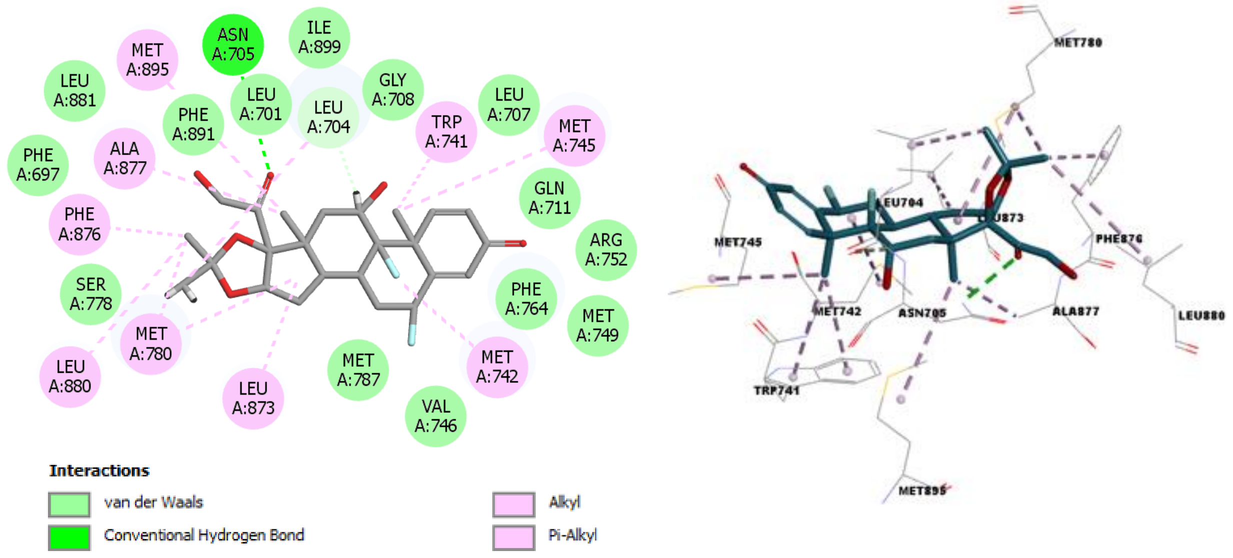

(**a**) (**b**)

Figure S6. Bioavailability radar graph. The ideal physicochemical region for oral bioavailability is the colored zone. LIPO (lipophilicity): −0.7 < XLOGP3 < +5.0; SIZE: 150 g/mol < MW < 500 g/mol; POLAR (polarity): 20 Å² < TPSA < 130 Å²; INSOLU (insolubility): −6 < LogS (ESOL) < 0; INSATU (in- saturation): 0.25 < Fraction Csp3 < 1; FLEX (flexibility): 0 < number. rotatable bonds < 9 [44].

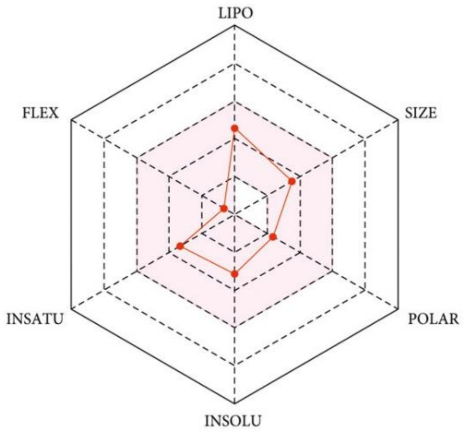

Figure S7. The bioavailability radar for the selected compounds and the reference molecule

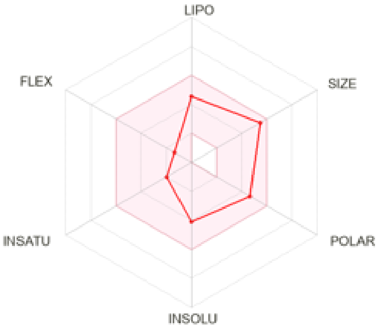

ZINC03977981

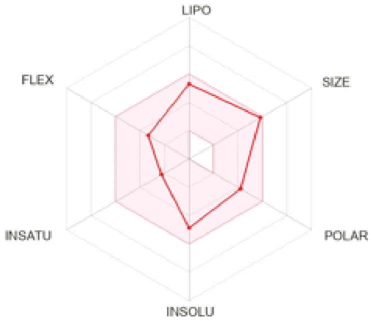

ZINC34176694

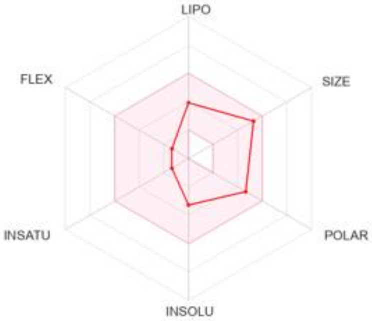

ZINC04097308

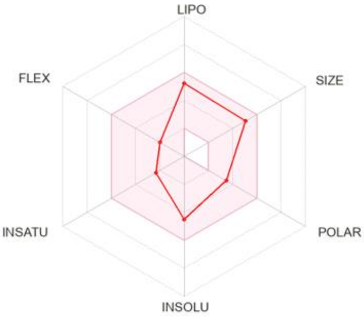

(CPA) Reference

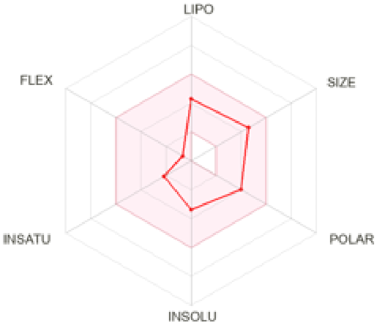

ZINC03876158

Figure S8. Predicted lipophilicity values (LogPo/w) of the compounds promising and the pivot compound.

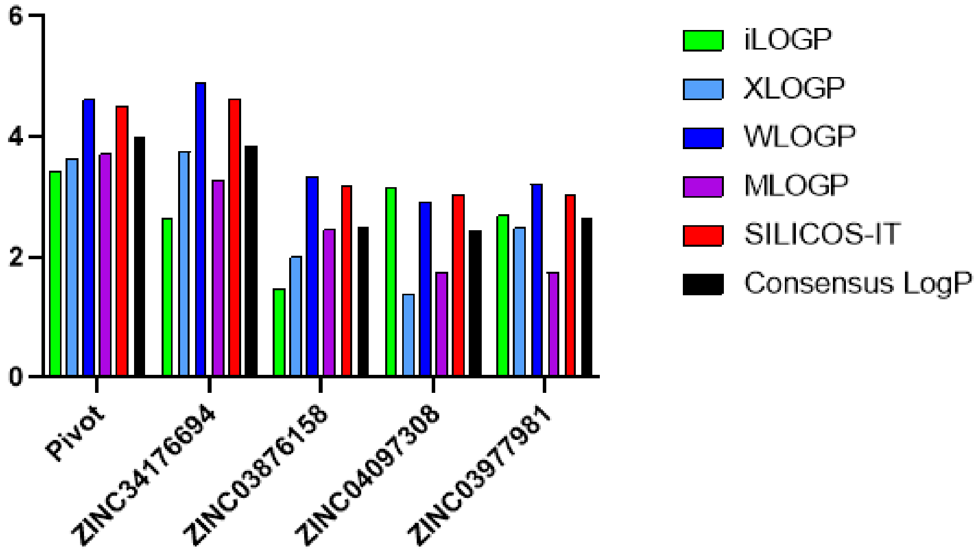

Figure S9. Predicted water solubility values (LogS) of the lead compounds and the pivot molecule.

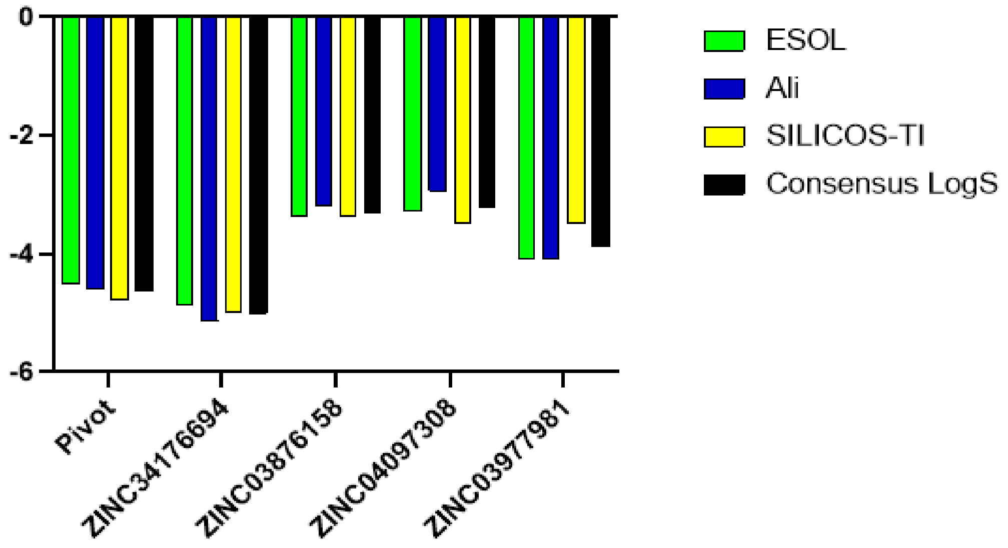

Supplement: Supplementary file 1 [file pharmaceuticals-18-00888-s001.zip › pharmaceuticals-3567176-supplementary.pdf]
